# Supplementary material for: Measuring women’s empowerment in aquaculture in northwestern Bangladesh using a project level women’s empowerment in fisheries index (pro-WEFI)
Source: PLoS One. 2025 Jan 10;20(1):e0314284. doi: 10.1371/journal.pone.0314284 (PMC11723601; doi:10.1371/journal.pone.0314284)
Supplement: S1 Questionnaires — (DOCX) [file pone.0314284.s002.docx]

**Questionnaire modules for pro-WEFI**

প্রো-ডাবলু.ই.এফ.আই. সার্ভের প্রশ্নপত্র

| This questionnaire should be administered separately to individuals identified as the **primary and secondary respondents**. You should complete the coversheet (Module G1) for each individual identified even if the individual is not available to be interviewed for reporting purposes.  এই প্রশ্নপত্রটি প্রাথমিক ও সহ-উত্তরদাতা হিসাবে চিহ্নিত ব্যক্তিদের পৃথকভাবে পরিচালনা করা উচিত। প্রতিটি ব্যক্তির জন্য কভারশিট (মডিউল জি 1) সম্পূর্ণ করা উচিত প্রতিবেদনের উদ্দেশ্যে, যদি কোনও ব্যক্তির সাক্ষাৎকার গ্রহণের জন্য উপলব্ধ না হয় তবেও।  Before starting the interview, please double check to ensure:  সাক্ষাৎকার শুরু করার আগে, দয়া করে আবার যাচাই করে নিশ্চিত করুন:  •You have completed identified the correct primary and/or secondary respondent(s);  আপনি সঠিক প্রাথমিক বা সহ-উত্তরদাতা (দের) সনাক্ত করেছেন।  •You have gained informed consent for the individual in the household questionnaire;  আপনি পরিবারের প্রশ্নাবলীর মধ্যে উত্তরদাতাকে সব বিষয় বুঝিয়ে সম্মতি অর্জন করেছেন।  •You have sought to interview the individual in private or where other members of the household cannot overhear or contribute answers.  আপনি সাক্ষাৎকারের জন্য এমন জায়গা বেছেছেন যেখানে একান্তে আপনি এই সাক্ষাৎকার নিতে পারবেন এবং পরিবারের অন্য সদস্যরা আপনাদের কথা শুনতে বা উত্তর দিতে পারবেন না ব্যক্তিগতভাবে বা যেখানে সেই ব্যক্তির নিতে চেয়েছেন।  •Do not attempt to make responses between the primary and secondary respondent the same—it is ok for them to be different.  প্রাথমিক এবং সহ-উত্তরদাতার উত্তর একই রকম করার চেষ্টা করবেন না — তাদের মতামত আলাদা হলে অসুবিধা নেই। |
| --- |

**Project Women’s Empowerment in Fisheries Index (মাছ চাষ ও ধরার ক্ষেত্রে মহিলাদের ক্ষমতা বৃদ্ধির সংকেত)**

*[Before beginning the interview it is necessary to introduce the household to the survey and obtain their consent to participate. Make it clear that participation in the survey is voluntary. Please read the following consent form to the respondent in the language of the interview.]* সাক্ষাৎকার শুরুর আগে সমীক্ষার বিষয়ে পরিবারকে বুঝানো এবং এই সমীক্ষায় তাঁদের অংশগ্রহণের জন্য সম্মতি নেয়া প্রয়োজন। এটি পরিষ্কার করে বোঝান যে এই সমীক্ষায় অংশগ্রহণ সম্পূর্ণ রূপে তাঁদের স্বেচ্ছায়। সাক্ষাৎকার যে ভাষায় হবে, উত্তরদাতাকে নিম্নলিখিত সম্মতি ফর্মটি সেই ভাষাতেই পড়ে শোনান।

*My name is ___________________ and I work for the WorldFish and Ecociate. We are conducting research to inform development of a survey tool to measure men’s and women’s empowerment in fisheries and aquaculture contexts. It will be used in World Fish programs in Bangladesh and other countries. We would like to request your participation in the questionnaire to help us test the questions to create the final survey.*

আমার নাম ……………………………………….……….. এবং আমি ওয়ার্ল্ড ফিশ এবং ইকোসিয়েটের জন্য কাজ করি। মাছ চাষের মাধ্যমে মাছ ও জলজ প্রাণী পালন প্রসঙ্গে পুরুষদের এবং মহিলাদের ক্ষমতা পরিমাপের জন্য একটি সমীক্ষা প্রশ্নপত্র বিকাশের জন্য আমরা গবেষণা করছি। এটি বাংলাদেশ এবং অন্যান্য দেশে ওয়ার্ল্ড ফিশ এর প্রোগ্রামগুলিতে ব্যবহৃত হবে। সমীক্ষার জন্য ফাইনাল প্রশ্নপত্রটা তৈরি করার উদ্দেশ্যে আপনাকে এই প্রশ্নপত্রের প্রশ্নগুলোর উত্তর দিতে অনুরোধ করছি।

*We would like to conduct an interview with a primary male or female decision maker in your household. The interview will take about 2 hours. Your participation in this survey is completely voluntary. If you choose to participate, you may refuse to answer certain questions or choose to stop participating at any time. There is no foreseen risk to your participation in this survey.*

আমরা আপনার পরিবারের কোনও মুখ্য পুরুষ বা মহিলা সিদ্ধান্ত গ্রহণকারীর সাথে এই সাক্ষাৎকার করতে চাই। সাক্ষাৎকারটি নিতে প্রায় 2 ঘন্টা সময় লাগবে। এই সমীক্ষায় আপনার অংশগ্রহণ সম্পূর্ণ রূপে স্বেচ্ছায়। আপনি যদি এতে অংশগ্রহণ করেন, তাহলে আপনি যে কোন প্রশ্নের উত্তর দিতে অস্বীকার করতে পারেন বা যে কোনও সময় অংশগ্রহন বন্ধ করতে পারেন। এই সমীক্ষায় আপনার অংশগ্রহণের কারণে আমরা ভবিষ্যতে আপনার জন্য কোনও ঝুঁকি দেখতে পাচ্ছিনা।

*We ask for your support by responding to the questions as honestly and fully as possible. Your responses will be kept completely confidential. We will not share the information that identifies you or your household members with anyone. This is not an assessment of your efforts and there are no right or wrong answers. Your responses will be used only for research purposes only, and all of the information obtained will be kept secure in our files. Your participation is very much appreciated.*

এই প্রশ্নগুলির যথাসম্ভব সততার সাথে এবং সম্পূর্ণরূপে জবাব দিয়ে আমরা আপনার সমর্থন চাইছি। আপনার উত্তরগুলি সম্পূর্ণ গোপনীয় রাখা হবে। আপনি বা আপনার পরিবারের সদস্যরা চিহ্নিত হতে পারেন এমন কোন তথ্য অন্য কোন ব্যক্তিকে আমরা জানাবো না। এটি আপনার জ্ঞান ও কার্যয়কলপের মূল্যায়ন নয় এবং এই প্রশ্নপত্রের প্রশ্নগুলোর কোনও ঠিক বা ভুল উত্তর নেই। আপনার উত্তরগুলো কেবল গবেষণার উদ্দেশ্যে ব্যবহার করা হবে এবং প্রাপ্ত সমস্ত তথ্য আমাদের ফাইলে সুরক্ষিত থাকবে। আপনি অংশগ্রহণ করলে আমরা বাধিত হব।

*A copy of this consent form as well as contact information will be provided to you if so desired. Do you have any questions? You can ask questions concerning the study, both before agreeing to be involved and during the course of the study.*

আপনি যদি চান তবে এই সম্মতি ফর্মের একটি কপি, এবং আমাদের যোগাযোগের তথ্য আপনাকে দেয়া হবে। আপনার কি এই বিষয়ে কিছু জিজ্ঞাস্য আছে? আপনি এই সমীক্ষাতে যুক্ত হওয়ার আগে এবং সাক্ষাতকার চলাকালীন এই সমীক্ষা সম্পর্কিত প্রশ্ন করতে পারেন।

*[Check whether the respondent has understood the informed consent and provide the respondent with a copy of the consent form.]*

*[উত্তরদাতা সম্মতি পত্রে উল্লিখিত বিষয়টি বুঝতে পেরেছেন কিনা তা নিশ্চিত করুন এবং উত্তরদাতাকে সম্মতি পত্রের একটি অনুলিপি প্রদান করুন।]*

**A1.** Did the respondent indicate voluntary consent to participate in the interview উত্তরদাতা সাক্ষাৎকার দেয়ার জন্য স্বেচ্ছায় সম্মতি জানিয়েছেন কি?

1 = yes হ্যাঁ

2 = no না >> end interview সাক্ষাৎকার সমাপ্ত করুন

**A2.** Are you a household member who is responsible for social and economic decision making related to fisheries, aquaculture and/or agriculture আপনি কি এই পরিবারের সদস্য হিসেবে মাছ চাষ ও কৃষিকাজ সম্পর্কিত সামাজিক ও অর্থনৈতিক সিদ্ধান্ত গ্রহণ করেন?

1 = yes হ্যাঁ >> Module G1 *মডিউল জি ১*

2 = no না

**A3.** Can you identify who in your household is one of the primary decision makers and can I speak with that person? আপনার পরিবারে মুখ্য সিদ্ধান্ত গ্রহণকারীকে কি আপনি চিহ্নিত পারবেন এবং আমি কি সেই ব্যক্তির সাথে কথা বলতে পারি?

1 = yes হ্যাঁ

2 = no না >> end interview সাক্ষাৎকার সমাপ্ত করুন

*Obtain consent with the new household member and proceed to Module G1.*

*নতুন পরিবারের সদস্যের সম্মতি গ্রহণ করার পর মডিউল জি১ এ যান।*

# MODULE G1 *মডিউল জি ১*. INDIVIDUAL IDENTIFICATION উত্তারদাতার ব্যক্তিগত পরিচয়

The **primary and secondary respondents** are those who are self-identified as the primary members responsible for decision making, both social and economic decisions related to agriculture, within the household.

মুখ্য উত্তরদাতা এবং সহ-উত্তরদাতা পরিবারের মধ্যে কৃষিক্ষেত্রের সাথে যুক্ত সামাজিক এবং অর্থনৈতিক দুই প্রকারের সিদ্ধান্ত গ্রহণের জন্য মুখ্য সদস্য হিসাবে নিজেদের পরিচিতি দিয়েছেন।

*A few notes to determine which household members they are:* উত্তরদাতা পরিবারের কি ধরনের সদস্য তা নির্ধারণ করার জন্য কয়েকটি ইঙ্গিত:

- *The primary and secondary respondents are usually husband and wife, however they can also be another member as long as there is one male and one female aged 18 and over (for instance a mother could be living with her adult son or father with an adult daughter).*

মুখ্য উত্তরদাতা এবং সহ-উত্তরদাতা সাধারণত স্বামী এবং স্ত্রী হয়ে থাকেন, তবে কিছু ক্ষেত্রে ১৮ বছর বা তার বেশি বয়সের পুরুষ এবং মহিলা থাকলে তাদের মধ্যে অন্য সম্পর্ক থাকতে পারে (উদাহরণস্বরূপ একজন মা তার প্রাপ্ত বয়স্ক ছেলের সাথে বা একজন প্রাপ্তবয়স্ক কন্যার তার বাবা সাথে থাকতে *পারেন)।*

- *In general, the primary decision maker is also the head of household, but this may not always be the case (i.e. elderly parent living with adult son/daughter and the adult son/daughter may be the primary or secondary respondent).*

*সাধারণভাবে, পরিবারের মুখ্য সিদ্ধান্ত গ্রহণকারী এবং পরিবারের প্রধান একই ব্যক্তি হন তবে এটি সবসময় নাও হতে পারে (অর্থাৎ বয়স্ক বাবা/মা যদি এবং প্রাপ্ত বয়স্ক পুত্র / কন্যার সাথে থাকেন ওনাদের যে কেউ প্রাথমিক বা গৌন উত্তরদাতা হতে পারেন)*

- *It may also be the case that there is only a primary female respondent and there is no adult male present in the household. In cases whereby the primary male adult is absent from the household due to male migration (has gone for work), and has been or is expected to be away for more than 6 months out of the next/previous 12 months, the primary female adult is considered the primary decision maker.*

*এমনও হতে পারে যে কেবলমাত্র একজন মহিলা উত্তরদাতা পরিবারে উপস্থিত আছেন এবং কোনও প্রাপ্তবয়স্ক পুরুষ উপস্থিত নেই। এমনও হতে পারে যে মুখ্য প্রাপ্তবয়স্ক পুরুষ কাজের জন্য অন্য জায়গায় থাকেন এবং বিগত বা আগামী ১২ মাসের মধ্যে ৬ মাস বা তার বেশি, পরিবারের সাথে থাকেননি বা থাকবেন না। এই ক্ষেত্রে, ওই মহিলাই প্রাপ্তবয়স্ক মুখ্য সিদ্ধান্ত নির্ধারক হিসাবে বিবেচিত হবেন।*

- *In cases where there is only a primary male and no female, do not interview the household; there must be a primary or secondary female in the household to administer the pro-WEAI.*

*এমন পরিবার যেখানে মুখ্য উত্তরদাতা হিসাবে কেবলমাত্র পুরুষ ব্যাক্তি উপস্থিত এবং কোন মহিলা উপস্থিত নেই, সেই পরিবারের সাক্ষাৎকার গ্রহণ করবেন না। প্রো-ডাব্লু.ই.এফ.আই.পরিচালনা করবার জন্য পরিবারে অবশ্যই মুখ্য অথবা সহ- উত্তরদাতা হিসেবে একজন মহিলা কে থাকতে হবে।*

| **Question প্রশ্ন** | **Code সংকেত** |  | **Question** **প্রশ্ন** | | **Code সংকেত** |
| --- | --- | --- | --- | --- | --- |
| **G1.01**. Household Identification:  **পারিবারিক পরিচয়** | \|  \|  \|  \|  \|  \|  \| \| --- \| --- \| --- \| --- \| --- \| --- \| |  | **G1.13.** Does respondent (or household head) agree to be contacted for research purposes that will not cost him/her any money?  **উত্তরদাতা (বা পরিবারের প্রধান) কি গবেষণার জন্য তার তথ্য সরবরাহ করতে রাজি ? এতে পরিবারের কোনও টাকা খরচ হবে না।** | | \|  \| \| --- \|   Yes হ্যাঁ ….1  No না…….2 |
| **G1.02.** Name of respondent currently being interviewed:  **উত্তরদাতার নাম: Surname, First name:**  **পদবি, নামের প্রথম অংশ** | \|  \|  \|  \|  \|  \|  \| \| --- \| --- \| --- \| --- \| --- \| --- \| |  | **G1.14.** Phone number of respondent (or household head) **উত্তরদাতার ফোন নম্বর (বা পরিবারের প্রধানের)** | |  |
| **G1.03.** Gender of respondent**:**  Man পুরুষ……1  Woman নারী.…2  **উত্তরদাতার লিঙ্গ:** | \|  \|  \|  \| \| --- \| --- \| --- \| |  | **~~G1.15.~~** ~~Outcome of interview (~~**~~Code 1~~**~~):~~  **~~সাক্ষাৎকারের ফলাফল (কোড ১)~~** | | Response auto captured in app |
| **G1.05.** Number of years respondent went to school  **কত বছর পর্যন্ত উত্তরদাতা স্কুলে গিয়েছিলেন** | \|  \|  \|  \|  \| \| --- \| --- \| --- \| --- \|  \|  \|  \| \| --- \| --- \| |  | **G1.16.** Ability to be interviewed alone (**Code 2**):  **একা সাক্ষাৎকার দেওয়ার ক্ষমতা (কোড ২)** | | \|  \| \| --- \| |
|  |  |  | **~~G1.17.~~** ~~Code of interviewer~~  **~~সাক্ষাৎকারকের কোড~~** | | Response auto captured in app |
| \|  \|  \|  \| \| --- \| --- \| --- \|   **G1.06.** Marital status of the respondent:  উত্তরদাতার বৈবাহিক অবস্থা:  Married বিবাহিত …………1 Single **অবিবাহিত**……4  Divorced বিবাহ বিচ্ছিন্ন………2 Separated আলাদা **থাকেন**……..5  Widowed বিধবা………3 Cohabiting **বিবাহ ছাড়াই সহাবস্থান করেন......6** | |  | **~~G1.18.~~** ~~Code of data entry~~  **~~তথ্য প্রবেশকের কোড~~** | | Not needed for digital surveys |
| **G1.07.** Religion of the respondent  **উত্তরদাতার ধর্ম**  **Ethnic group code ধর্মের কোড**  Islam **ইসলাম** …………1 Hindu  **হিন্দু**……2  Christian **খ্রিষ্টান**………3 Others (Specify) **অন্য** (**বিবরণ দিন)** …..4 | \|  \|  \|  \| \| --- \| --- \| --- \| |  | | **Code 1 (কোড ১)**  Completed সম্পূর্ণ …………………...1  Household member too ill to respond/ Cognitively impaired পরিবারের সদস্য অসুস্থ সাক্ষাৎকার দিতে পারবেন না / মানসিক প্রতিবন্ধকতা আছে……………………………………………….2  Respondent not at home/Temporarily unavailable **উত্তরদাতা সাময়িক ভাবে বাড়িতে নেই** ………………..3  Respondent not at home/ Extended absence **উত্তরদাতা অনেক দিন ধরে বাড়িতে নেই** ………….4  Refused **সাক্ষাৎকার দিতে রাজি হননি…**……………..…5  Could not locate **উত্তরদাতার বাড়ি** সনাক্ত করা যায়নি…..…6  **Code 2 (কোড ২)**  Alone একা …………………………………..1  With adult women present প্রাপ্তবয়স্ক মহিলার উপস্থিতিতে.............................................2  With adult men present প্রাপ্ত বয়স্ক পুরুষের উপস্থিতিতে ...........................................3  With adults mixed gender present প্রাপ্তবয়স্ক পুরুষ এবং মহিলাদের উপস্থিতিতে...4  With children present শিশুর উপস্থিতিতে …………………………………..5  With adults mixed gender and children present প্রাপ্তবয়স্ক পুরুষ এবং মহিলা ও শিশুর উপস্থিতিতে ..…..6 | |
| **G1.08.** Language of the interview **সাক্ষাৎকারের ভাষা** |  |  |  |  |  |
|  |  |  |  |  |  |
| **G1.11.** Type of household **পরিবারের ধরণ**  Man and woman adult **প্রাপ্তবয়স্ক পুরুষ এবং মহিলা দুজনেই আছেন .........**1  Woman adult only **শুধু প্রাপ্তবয়স্ক মহিলা আছেন ..............**2  Man adult only **শুধু প্রাপ্তবয়স্ক** পুরুষ **আছেন ...........**3 🡪 *Do not administer (সাক্ষাৎকার পরিচালনা করবেন না)  Polygamous **একটির বেশি স্বামী/স্ত্রী আছেন** (**বহু-বিবাহিত) ....**4 | \|  \|  \|  \| \| --- \| --- \| --- \| |  |  |  |  |
| **G1.12.** Type of respondent **উত্তরদাতার প্রকার**  Primary man প্রধান পুরুষ 1  Primary woman প্রধান **মহিলা** 2 | \|  \|  \| \| --- \| --- \| |  |  |  |  |

*Please try to conduct the interview alone and without children or other male or female adults present*

দয়া করে সাক্ষাৎকারটি কোন শিশু বা অন্যান্য প্রাপ্তবয়স্ক পুরুষ অথবা মহিলাদের অনুপস্থিতিতে, একা পরিচালনা করার চেষ্টা করুন।

## MODULE G2: Power/input in household decision-making around production and income generation

**মডেল জি 2. উৎপাদন এবং আয়ের উপর পরিবারের সিদ্ধান্ত গ্রহণের ক্ষমতা**

| Now I’d like to ask you some questions about your participation in certain types of work activities and on making decisions on various aspects of household life.  **~~age~~**  নির্দিষ্ট ধরণের কাজে আপনার অংশগ্রহণ এবং গৃহস্থালীর জীবনের বিভিন্ন দিক নিয়ে সিদ্ধান্ত নেওয়ার বিষয়ে আমি আপনাকে কিছু প্রশ্ন জিজ্ঞাসা করতে চাই। | | Did you yourself (singular) participate in [ACTIVITY] in the past 12 months (that is during the last [one/two] cropping seasons), from [PRESENT MONTH] last year to [PRESENT MONTH] this year?  ***আপনি কি নিজে (একক) গত ১২ মাসে (যা গত এক / দুই ফসলের মরসুমের সময়কালের সমান) গত বছর থেকে এই বছরে [চলতি মাস পর্যন্ত] এই কাজে যোগ দিয়েছেন?***  Yes***হ্যাঁ***…..1  No***না***…..2 **🡪**  NEXT ACTIVITY  ***পরবর্তী কার্য্যকলাপ*** | When decisions are made regarding [ACTIVITY], who is it that is normally involved in making the decision?  ***যখন [এই কাজের*] *বিষয়ে সিদ্ধান্ত নেওয়া হয়, তখন কে /কারা সাধারণতঃ এই সিদ্ধান্ত নেয়ায় জড়িত থাকেন?***  **ENTER UP TO THREE CODES**  *তিনটি পর্যন্ত কোড ব্যবহার করা যাবে*  **Code 3**  **IF RESPONSE IS SELF ONLY (OPTION 1) 🡪**  *যদি প্রতিক্রিয়া কেবল নিজে হয়* **(OPTION 1)**  **G2.06** | | | How much input did you yourself (singular) have in making decisions about [ACTIVITY]?  **[*এই কাজের*]** ***বিষয়ে সিদ্ধান্ত নেওয়ার ক্ষেত্রে আপনি নিজে (একক) কতটা যোগদান (মতামত) করেছিলেন?***  **Code 5** | To what extent do you feel you yourself (singular) can participate in decisions regarding [ACTIVITY] if you want(ed) to?  ***আপনি যদি চান তবে নিজে (একা) কোন পর্যন্ত* [*এই কাজের*]** ***বিষয়ে সিদ্ধান্তে অংশ নিতে পারেন বলে মনে করেন?***  **Code 4** | If there was disagreement in decisions regarding [ACTIVITY], whose opinion usually prevailed?  **[*এই কাজের*]** ***বিষয়ে সিদ্ধান্তে যদি মতবিরোধ হয়, তবে কার মতামত সাধারণত প্রাধান্য পায়?***  **Code 3** | How much input did you have in decisions about how much of the outputs of [ACTIVITY] to keep for consumption at home rather than selling?  **[*এই কাজের*] থেকে উৎপাদন *এর কতটা বিক্রি না করে ঘরে রাখবেন সে সম্পর্কে আপনার নিজের (একক) কতটা যোগদান (মতামত) ছিল?***  **Code 5** | How much input did you yourself have in decisions about how to use of income generated from [ACTIVITY]?  **[*এই কাজের*] *থেকে যে টাকা আয় হয় সেটা কীভাবে ব্যবহার করবেন সে বিষয়ে সিদ্ধান্তে আপনার নিজের (একক) কতটা যোগদান (মতামত) ছিল*?**  **Code 5** |  |
| --- | --- | --- | --- | --- | --- | --- | --- | --- | --- | --- | --- |
| **ACTIVITY** | | **G2.01** | **G2.02** | | | **G2.03** | **G2.04** | **G2.05** | **G2.06** | **G2.07** |  |
|  |  |  | **#1** | **#2** | **#3** |  |  |  |  |  |  |
| **FISHING/AQUACULTURE মাছ বা অন্য জলজ প্রাণী চাষ** | | | | | | | | | | | |
| **A** | Fish farming homestead pond বাড়ির পুকুরে মৎস্য চাষ |  |  |  |  |  |  |  |  |  |  |
| **B** | Fish farming commercial pond বাণিজ্যিক পুকুরে মাছ চাষ |  |  |  |  |  |  |  |  |  |  |
| **C** | Off shore fishing গভীর সমুদ্রের মাছ ধরা |  |  |  |  |  |  |  |  |  |  |
| **D** | In shore fishing অগভীর সমুদ্রের মাছ ধরা |  |  |  |  |  |  |  |  |  |  |
| **E** | Shore fishing নদী/খালের মাছ ধরা |  |  |  |  |  |  |  |  |  |  |
| **POST FISHING মাছ ধরার পরবর্তি কাজ** | | | | | | | | | | | |
| **F** | Fish processing / drying মৎস প্রক্রিয়াজাতকরণ / শুকানো |  |  |  |  |  |  |  |  |  |  |
| **G** | Fish storage মৎস সংরক্ষণ করার কাজ |  |  |  |  |  |  |  |  |  |  |
| **H** | Fish marketing and retailing (transportation/ selling/ trading of fish) মৎস বিপণন খুচরা বিক্রয় (মাছ পরিবহন / বিক্রয় / বাণিজ্য) |  |  |  |  |  |  |  |  |  |  |
| **CROPS AND LIVESTOCK শস্য ও প্রানী সম্পদ** | | | | | | | | | | | |
| **I** | Food crop farming (i.e. types of crops to grow for consumption) খাদ্য শস্য (খাবার উদ্যেশ্যে যা ফলান হয়। যেমন - ধান, গম ) |  |  |  |  |  |  |  |  |  |  |
| **J** | Cash crop farming (i.e. types of crops to grow for selling in the market) অর্থকরী ফসল (যে ফসল বিক্রির উদ্যেশ্যে ফলান হয়। যেমন – পাট) |  |  |  |  |  |  |  |  |  |  |
| **K** | Large livestock raising (cattle, buffaloes) and processing of milk or meat বৃহৎ প্রানীজ সম্পদ (গোরু , মহিষ ) যা দুধ ও মাংসের জন্য পোষা হয় |  |  |  |  |  |  |  |  |  |  |
| **L** | Small Livestock raising (sheep, goats) and processing of milk or meat ক্ষুদ্র প্রানীজ সম্পদ (ছাগল, ভেড়া) যা দুধ ও মাংসের জন্য পোষা হয় |  |  |  |  |  |  |  |  |  |  |
| **M** | Poultry and other animals raising (chickens, ducks, turkeys) and processing of eggs and/or meat পোল্ট্রি এবং অন্যান্য (মুরগী, হাঁস, টার্কি) যা ডিম ও মাংসের জন্য পালন হয় |  |  |  |  |  |  |  |  |  |  |
| **OTHER ACTIVITIES অন্যান্য কাজকর্ম** | | | | | | | | | | | |
| **N** | Non-farm economic activities: Small business, self-employment, buy-and-sell খামারহীন অর্থনৈতিক কার্য: ছোট ব্যবসা, স্ব-কর্মসংস্থান, কেনা বেচা |  |  |  |  |  |  |  |  |  |  |
| **O** | Wage and salary employment: in-kind or monetary work both agriculture and other wage work মজুরি ও বেতন কর্মসংস্থান: আর্থিক কাজ - কৃষি এবং অন্যান্য উভয় মজুরির কাজ |  |  |  |  |  |  |  |  |  |  |
| **P** | Large, occasional household purchases (bicycles, land transportation vehicles) বড়, মাঝে মাঝে পরিবারের ক্রয় (সাইকেল, ভুমি পরিবহন যানবাহন) |  |  |  |  |  |  |  |  |  |  |
| **Q** | Routine household purchases (food for daily consumption or other household needs) দৈনন্দিন পরিবারের ক্রয় (খাদ্য সামগ্রী বা অন্যান্য পরিবারের নিত্য প্রয়োজনীয় দ্রব্য) |  |  |  |  |  |  |  |  |  |  |

| **Code 3 কোড 3** | **Code 4 কোড 4** | **Code 5 কোড 5** |
| --- | --- | --- |
| Self নিজে …………1  Partner/Spouse in HH পরিবারে বসবাসকারী সঙ্গী/স্বামী/স্ত্রী …………2  Partner/Spouse outside HH পরিবারের থেকে দূরে থাকা সঙ্গী/স্বামী/স্ত্রী …………3  Other HH member পরিবারের অন্য সদস্য…………………4  NON-HH, NON-FAMILY MEMBER পরিবারের বাইরে থাকা ব্যক্তি যে পরিবারের সদস্য নন 5  Non-hh, FAMILY MEMBER পরিবারের বাইরে থাকা ব্যক্তি যে পরিবারের সদস্য 6  NOT APPLICABLE প্রযোজ্য নয়/ সিদ্ধান্ত নেওয়া হয়নি……….98 >> NEXT Row পরের বিষয় | Not at all একটুও নয়…1  Small extent অল্প পরিমাণ...2  Medium extent মাঝারি পরিমাণ...3  To a high extent উচ্চ মাত্রায় …4 | Little to no input in decisions সিদ্ধান্তে মতামত নেই/ অতিসামান্য…………..1  Some input in decisions সিদ্ধান্তে মতামত অতি সামান্য…………………..2  Input into most or all decisions সমস্ত সিদ্ধান্তে সর্বাধিক মতামত…………….3  Not applicable / no decision made প্রযোজ্য নয় / সিদ্ধান্ত নেওয়া হয়নি……98 |

## MODULE G3 (A): Access to and control over productive capital (LAND and POND) *উৎপাদনশীল মূলধনের উপর আয়ত্ত ও নিয়ন্ত্রণ (* জমি ও পুকুর *)*

| Now I’d like to ask you specifically about your household’s land and ponds. এখন আমি আপনাকে আপনার পরিবারের জমি এবং পুকুর সম্পর্কে বিশেষভাবে জিজ্ঞাসা করতে চাই। | | | |
| --- | --- | --- | --- |
| **QUESTION** | **RESPONSE** | | |
| **G3.01.** Does anyone in your household currently own or cultivate land? This could be land that you have access to without legal ownership or land that you legally own or lease. **আপনার পরিবারের কেউ কি বর্তমানে চাষ জমির মালিক বা চাষ করেন? এই জমি আপনার নিজের হতে পারে অথবা ভাড়া নেওয়া হতে পারে অথবা আপনার দখলে আইনত মালিকানা বিহিত জমি হতে পারে।** | YES **হ্যাঁ**.......1  NO **না**.........2**→G3.03** | | |
| **G3.02.** Do you own any of the land owned or cultivated by your household? **আপনার পরিবারের চাষাবাদ করা কোনও জমির আপনি কি মালিক?**  **CIRCLE ONE** একটিকে *বৃত্ত চিহ্ন দিন* | YES, SOLELY **হ্যাঁ,** কেবলমাত্র **নিজে** ............................. 1  YES, JOINTLY **হ্যাঁ যৌথভাবে**. …………………………2  YES, SOLELY AND JOINTLY **হ্যাঁ, একক ও যৌথভাবে** ...... 3  NO **না**....................................................................... 4 | | |
| **G3.03.** Does anyone in your household currently own or cultivate ponds? **আপনার পরিবারের কেউ কি বর্তমানে পুকুরের মালিক বা চাষ করেন?** | YES **হ্যাঁ**........1  NO **না**..........2→***G3.05*** | | |
| **G3.04.** Who generally makes decisions about what to grow in this/these pond(s)? **এই পুকুর/ পুকুরগুলিতে কী চাষ করা উচিত/ হবে সে সম্পর্কে সাধারণত কে সিদ্ধান্ত নেন?**  **Code 3 কোড 3: ENTER UP TO THREE CODES** *তিনটি পর্যন্ত কোড ব্যবহার করা যাবে* | **#1** | **#2** | **#3** |
|  |  |  |  |
| **G3.05.** Do you [NAME] solely or jointly cultivate any ponds? **আপনি কি একা বা যৌথভাবে কোনও পুকুর চাষ করেন?**  **CIRCLE ONE** একটিকে *চিহ্নত করুন* | YES, SOLELY **হ্যাঁ,** কেবল **নিজে** ............................. 1  YES, JOINTLY **হ্যাঁ যৌথভাবে**. …………………………2  YES, SOLELY AND JOINTLY **হ্যাঁ, একক ও যৌথভাবে** ...... 3  NO **না**....................................................................... 4 | | |
| G3.06. Who generally makes decisions about what to grow in the ponds that you yourself cultivate? **যে পুকুর এ আপনি নিজে চাষ করেন, সেই পুকুরে কী চাষ করবেন সে সম্পর্কে কে সাধারণত সিদ্ধান্ত নেয়?**  **Code 3 কোড 3: ENTER UP TO THREE CODES** *তিনটি পর্যন্ত কোড ব্যবহার করা যাবে* | **#1** | **#2** | **#3** |
|  |  |  |  |
| **G3.7.** Do you own any of the ponds owned or cultivated by your household? **আপনার পরিবারের মালিকানা থাকা বা চাষ করা কোন পুকুরের মালিক কি আপনি নিজে?**  **CIRCLE ONE** একটিকে *চিহ্নত করুন* | YES, SOLELY **হ্যাঁ,** কেবলমাত্র **নিজে** ............................. 1  YES, JOINTLY **হ্যাঁ যৌথভাবে**. …………………………2  YES, SOLELY AND JOINTLY **হ্যাঁ, একক ও যৌথভাবে** ...... 3  NO **না**....................................................................... 4 | | |
| **G3.8.** Does anyone in your household currently access fishing grounds or gleaning areas (common areas where you can gather and collect in shallow water or exposed land)? **আপনার পরিবারের কেউ কি মাছ ধরার জন্য এমন জলা জায়গায় যান যেখানে সর্বসাধারণে যেতে পারে?** (**সর্বসাধারণের ব্যবহারযোগ্য জায়গা যেখান থেকে মাছ সংগ্রহ করা যেতে পারে - যেমন অগভীর জলা জমি)** | YES **হ্যাঁ**........1  NO **না**.........2→ ***G3.10*** | | |
| **G3.9.** Do you access any of the fishing grounds or gleaning areas (common areas where you can gather and collect in shallow water or exposed land)? **আপনি কেউ কি মাছ ধরার জন্য এমন জলা জায়গায় যান যেখানে সর্বসাধারণে যেতে পারে?** (**সর্বসাধারণের ব্যবহারযোগ্য জায়গা যেখান থেকে মাছ সংগ্রহ করা যেতে পারে - যেমন অগভীর জলা জমি**  **CIRCLE ONE** একটিকে *চিহ্নত করুন* | YES, SOLELY **হ্যাঁ,** কেবলমাত্র **নিজে** ............................. 1  YES, JOINTLY **হ্যাঁ যৌথভাবে**. …………………………2  YES, SOLELY AND JOINTLY **হ্যাঁ, একক ও যৌথভাবে** ...... 3  NO **না**....................................................................... 4 | | |

| **Code 3** |
| --- |
| Self নিজে …………1  Partner/Spouse in HH পরিবারে বসবাসকারী সঙ্গী/স্বামী/স্ত্রী …………2  Partner/Spouse outside HH পরিবারের থেকে দূরে থাকা সঙ্গী/স্বামী/স্ত্রী …………3  Other HH member পরিবারের অন্য সদস্য…………………4  NON-HH, NON-FAMILY MEMBER পরিবারের বাইরে থাকা ব্যক্তি যে পরিবারের সদস্য নন 5  Non-hh, FAMILY MEMBER পরিবারের বাইরে থাকা ব্যক্তি যে পরিবারের সদস্য 6  Don’t know জানা নেই..........................................................................96  NOT APPLICABLE প্রযোজ্য নয়/ সিদ্ধান্ত নেওয়া হয়নি……….98 |

MODULE G3 (A)-cont: Access to and control over productive capital (ASSETS other than LAND and POND) **উৎপাদনশীল মূলধনের উপর আয়ত্ত (অ্যাক্সেস) এবং নিয়ন্ত্রণ (জমি এবং পুকুর ব্যতীত অন্যান্য সম্পদ)**

| Now I’d like to ask you about a number of items that could be used to generate income. এখন আমি আপনাকে আইটেমগুলির সংখ্যা সম্পর্কে জিজ্ঞাসা করতে চাই যা আয় তৈরিতে ব্যবহৃত হতে পারে। | | Does anyone in your household currently have any [ITEM]? আপনার পরিবারের কারও কাছে বর্তমানে কি এই [জিনিস গুলো] আছে?  Yes **হ্যাঁ** ……………1  No **না** ……..2 **>> NEXT [ITEM] পরের** [জিনিস] | Do you [NAME] own any [ITEM]?  আপনার নিজের নামে কি এই [জিনিস গুলো] আছে?  YES, SOLELY **হ্যাঁ,** কেবল **নিজে** ........................... 1  YES, JOINTLY **হ্যাঁ যৌথভাবে**. …………2  YES, SOLELY & JOINTLY **হ্যাঁ, একক ও যৌথভাবে** ........ 3  NO **না** .................................... 4 |
| --- | --- | --- | --- |
| **ITEM** আইটেম | | **G3.10** | **G3.11** |
|  |  |  |  |
| **FISHING/AQUACULTURE মৎস্য চাষ /** **জলজ চাষ** | | | |
| **A** | Large fish (e.g. mature rohu, tilapia, etc.) বড় মাছ (উদাহরন -রুই, তেলাপিয়া) |  |  |
| **B** | Small fish (e.g. mola, other small indigenous species) ছোট মাছ (উদাহরন -মৌরলা, **অন্যান্য ছোট দেশীয় প্রজাতি**) |  |  |
| **C** | Fish producing equipment (e.g. fish feed, medicine, other inputs including lime for ponds, etc) **মাছ উৎপাদন সরঞ্জাম (উদাঃ মাছের খাবার, ওষুধ, পুকুরের জন্য চুন সহ অন্যান্য উপকরণ ইত্যাদি)** |  |  |
| **D** | Fish processing equipment (e.g. drying mats, knives**) মৎস পক্রিয়াকরণ সরঞ্জাম (উদাঃ শুকানোর মাদুর, ছুরি)** |  |  |
| **E** | Fish storage equipment (e.g. sacks, bundles, freezers) **মৎস** সংরক্ষণ **সরঞ্জাম (উদাঃ বস্তা, বান্ডিল, ফ্রিজার)** |  |  |
| **F** | Fish transportation equipment (e.g. canoe or boat for fishing) **মাছ পরিবহনের সরঞ্জাম (যেমন মাছ ধরার জন্য নৌকা বা** ডোঙ্গা**)** |  |  |
| **G** | Fishing and harvesting equipment (e.g. nets, line and hooks) **মাছ ধরা ও সংগ্রহের সরঞ্জাম (যেমন জাল, রশি এবং** আঙটা**/হুক)** |  |  |
| **CROPS AND LIVESTOCK** শস্য ও প্রানীজ সম্পদ | | | |
| **H** | Large livestock (oxen, cattle) বৃহৎ প্রানী সম্পদ (**বলদ, গবাদি পশু**) |  |  |
| **I** | Small livestock (goats, sheep) **ছোট পশুপাল (ছাগল, ভেড়া)** |  |  |
| **J** | Chickens, Ducks, Turkeys, Pigeons **মুরগী, হাঁস, টার্কি, কবুতর** |  |  |
| **K** | Mechanized farm equipment (tractor-plough, power tiler, treadle pump) **যান্ত্রিক খামার সরঞ্জাম (ট্র্যাক্টর-লাঙল, পাওয়ার টিলার, ট্রেডল পাম্প)** |  |  |
| **L** | Non-mechanized farm equipment (hand tools, animal drawn plough) **যান্ত্রিকীকরণহীন খামার সরঞ্জাম (হাত সরঞ্জাম, প্রাণী আঁকার লাঙ্গল)** |  |  |
| **OTHER অন্যান্য** | | | |
| **M** | Nonfarm business equipment **খামারহীন অর্থনৈতিক কার্যক্রম ব্যবসায়ের সরঞ্জাম** |  |  |
| **N** | House (and other structures) **ঘর (এবং অন্যান্য কাঠামো)** |  |  |
| **O** | Large consumer durables (fridge, TV, sofa) **বৃহত্তর গ্রাহক টেকসই (ফ্রিজ, টিভি, সোফা)** |  |  |
| **P** | Small consumer durables (radio, cookware) **ক্ষুদ্র গ্রাহক টেকসই (রেডিও, কুকওয়্যার)** |  |  |
| **Q** | Means of communication (e.g. cell phone, telephone) **যোগাযোগের মাধ্যম (উদাঃ মুঠোফোন, টেলিফোন)** |  |  |
| **R** | Internet **ইন্টারনেট** |  |  |
| **S** | Other land not used for agricultural purposes (pieces, residential or commercial land) **অন্যান্য জমি, কৃষিকাজের জন্য ব্যবহৃত হয় না (টুকরো, আবাসিক বা বাণিজ্যিক জমি)** |  |  |
| **T** | Means of transportation (bicycle, motorcycle, car) **পরিবহণের উপায় (সাইকেল, মোটরসাইকেল, গাড়ি)** |  |  |

## MODULE G3 (B): Access to and control over Financial Services

| Now I’d like to ask about your household’s experience with borrowing money or other items (in-kind) in the past 12 months.  এবার আমি গত ১২ মাসে ঋন (নগদ বা জিনিস) গ্রহণের বিষয়ে আপনার পারিবারের অভিজ্ঞতা সম্পর্কে জিজ্ঞাসা করতে চাই। | | Would you or anyone in your household be able to take a loan or borrow cash/in-kind from [SOURCE] if they wanted to?  আপনি বা আপনার পরিবারের কেউ চাইলে কি এই জায়গা থেকে ঋণ (নগদ বা জিনিস) নিতে পারবেন?  Yes **হ্যাঁ** …………..1  No **না**……………2 **🡪 NEXT SOURCE পরবর্তী জায়গা**  Maybe বোধহয়……………3 | Has anyone in your household taken any loans or borrowed cash/in-kind from [SOURCE] in the past 12 months, from [PRESENT MONTH] last year to [PRESENT MONTH] this year?  আপনার পরিবারের কেউ কি গত ১২ মাসে [গত বছরের [এই মাস] থেকে এই বছরের উপস্থিত মাস অবধি] এই জায়গা থেকে ঋণ (নগদ বা এই ধরনের) নিয়েছেন?  Yes, cash **হ্যাঁ, নগদ** 1 **🡪 G3.15**  Yes, in-kind **হ্যাঁ, জিনিস**  2 **🡪 G3.15**  Yes, cash and in-kind **হ্যাঁ, নগদ এবং এই ধরনের** 3 **🡪 G3.15**  No 4  Don’t know জানি না ………………….97 **🡪 NEXT SOURCE পরবর্তী জায়গা** | Why did not you take any loans or borrowed cash/in-kind from [SOURCE] in the past 12 months, from [PRESENT MONTH] last year to [PRESENT MONTH] this year?  আপনি কেন গত ১২ মাসে [গত বছরের [এই মাস] থেকে এই বছরের উপস্থিত মাস অবধি] এই জায়গা থেকে ঋণ (নগদ বা জিনিস) নেন নি?  **Code 5**  **কোড 5**  **🡪 NEXT SOURCE পরবর্তী জায়গা** | Who made the decision to borrow from [SOURCE] most of the time?  কে বেশিরভাগ সময় এই জায়গা থেকে ঋণ নেওয়ার সিদ্ধান্ত নিয়েছিলেন?  **ENTER UP TO THREE CODES**  *তিনটি পর্যন্ত কোড ব্যবহার করা যাবে*  **Code 3**  **কোড 3** | | | Who makes the decision about what to do with the money/ item borrowed from [SOURCE] most of the time?  এই জায়গা থেকে ঋণ নেওয়া টাকা বা জিনিস কীভাবে ব্যবহার করা হবে সে সিদ্ধান্ত বেশিরভাগ সময় কে নেয়?  **ENTER UP TO THREE CODES**  *তিনটি পর্যন্ত কোড ব্যবহার করা যাবে*  **Code 3**  **কোড 3** | | | Who is responsible for repaying the money or item borrowed from [SOURCE]?  এই জায়গা থেকে ঋণ নেওয়া টাকা বা জিনিস শোধ করার দায়িত্ব কার?  **ENTER UP TO THREE CODES**  *তিনটি পর্যন্ত কোড ব্যবহার করা যাবে*  **Code 3**  **কোড 3** | | |
| --- | --- | --- | --- | --- | --- | --- | --- | --- | --- | --- | --- | --- | --- |
| **LENDING SOURCES ঋণ নেবার জায়গা** | | **G3.12** | **G3.13** | **G3.14** | **G3.15** | | | **G3.16** | | | **G3.17** | | |
|  |  |  |  |  | **#1** | **#2** | **#3** | **#1** | **#2** | **#3** | **#1** | **#2** | **#3** |
| **A** | Non-governmental organization  বেসরকারী প্রতিষ্ঠান  (NGO)  (এনজিও) |  |  |  |  |  |  |  |  |  |  |  |  |
| **B** | Informal lender  অনানুষ্ঠানিক ঋণদানকারী |  |  |  |  |  |  |  |  |  |  |  |  |
| **C** | Formal lender (bank/financial institution)  আনুষ্ঠানিক ঋণদানকারী (ব্যাংক / আর্থিক প্রতিষ্ঠান) |  |  |  |  |  |  |  |  |  |  |  |  |
| **D** | Friends or relatives  বন্ধু বা আত্মীয়স্বজন |  |  |  |  |  |  |  |  |  |  |  |  |
| **E** | Group based micro-finance or lending including VSLAs / SACCOs / merry-go-rounds  গ্রুপ ভিত্তিক মাইক্রো ফিনান্স বা ঋণ প্রদান সহ ভিএসএলএ / স্যাকসিও / ম্যারি-গো-রাউন্ড |  |  |  |  |  |  |  |  |  |  |  |  |
| **F** | Others (any context specific sources)  অন্যান্য (কোনও প্রসঙ্গ নির্দিষ্ট উৎস) |  |  |  |  |  |  |  |  |  |  |  |  |

| **G3.18** | **Code 3** | **Code 5** |
| --- | --- | --- |
| An account can be used to save money, to make or receive payments, or to receive wages or financial help. Do you, either by yourself or together with someone else, currently have an account at any of the following places: a bank or other formal institution?  ***কোনও অ্যাকাউন্ট অর্থ সঞ্চয় করতে, অর্থ প্রদান করতে বা গ্রহণ করতে, বা মজুরি বা আর্থিক সহায়তা পেতে ব্যবহার করা যেতে পারে।***  ***আপনার নিজের দ্বারা বা অন্য কারও সাথে একত্রে, বর্তমানে নিম্নলিখিত কোনও স্থানে অ্যাকাউন্ট রয়েছে:***  ***ব্যাংক বা অন্য কোনও প্রতিষ্ঠান?***  Yes **হ্যাঁ** ……………………..1 **🡪 NEXT MODULE পরবর্তী মডিউল**  No **না** ……………………….2  Don’t’ know জানি না ….97 | Self নিজে …………1  Partner/Spouse in HH পরিবারে বসবাসকারী সঙ্গী/স্বামী/স্ত্রী …………2  Partner/Spouse outside HH পরিবারের থেকে দূরে থাকা সঙ্গী/স্বামী/স্ত্রী …………3  Other HH member পরিবারের অন্য সদস্য…………………4  NON-HH, NON-FAMILY MEMBER পরিবারের বাইরে থাকা ব্যক্তি যে পরিবারের সদস্য নন 5  Non-hh, FAMILY MEMBER পরিবারের বাইরে থাকা ব্যক্তি যে পরিবারের সদস্য 6  NOT APPLICABLE প্রযোজ্য নয়/ সিদ্ধান্ত নেওয়া হয়নি……….98 | Have enough money, so did not need any loan পর্যাপ্ত অর্থ আছে, তাই কোনও ঋণের দরকার পড়েনি……………………………………...1  Afraid of losing collateral জামানত হারাতে ভয় পায় ……………….2  Do not have enough collateral/did not qualify for the loan পর্যাপ্ত জামানত নেই / ঋণের জন্য যোগ্যতা অর্জন করেনি…………………….3  Afraid cannot pay back the money আতঙ্কিত, টাকা ফেরত দিতে পারবে না ….4  Interest rate/other costs too high সুদের হার / অন্যান্য খরচ খুব বেশি …….5  Not allowed to borrow/family dispute in borrowing decision ঋণ গ্রহণের সিদ্ধান্তে পারিবারিক বিরোধ / ঋণ গ্রহণের অনুমতি নেই…………....6  Place of lender is too far ঋণদাতার জায়গাটি খুব দূরে ……….7  Religeous reasons ধর্মের কারণে ……….8  Other, specify অন্যান্য, নির্দিষ্ট ………………………………….9 |

MODULE G3C: Access to INFORMATION AND EXTENSION SERVICES তথ্য এবং কৃষি প্রযুক্তি সম্পর্কিত পরিষেবার জায়গা গুলোতে পৌঁছোবার ক্ষমতা

|  | | Has anyone in your household received any technical advice or information on [TOPIC] in the past year, from [PRESENT MONTH] last year to [PRESENT MONTH] this year?  আপনার পরিবারে কেউ কি গত বছর [বর্তমান মাস] থেকে এই বছরের [উপস্থাপিত মাস] অবধি [এই বিষয়ে] কোনও প্রযুক্তিগত পরামর্শ বা তথ্য পেয়েছেন?  Yes **হ্যাঁ** ……………………..1  No **না** ……………..….2 **🡪 NEXT TOPIC পরবর্তী বিষয়** | Did you yourself (singular) receive any technical advice or information on [TOPIC] in the past year, from [PRESENT MONTH] last year to [PRESENT MONTH] this year?  আপনি নিজে (একক) গত বছর [বর্তমান মাস] থেকে এই বছরের [উপস্থাপিত মাস] অবধি [এই বিষয়ে] কোনও প্রযুক্তিগত পরামর্শ বা তথ্য পেয়েছেন?  Yes **হ্যাঁ** ……………………..1  No **না** ……………..….2 **🡪 NEXT TOPIC পরবর্তী বিষয়** | How well were you able to act on/implement advice and information on [TOPIC] from?  এই বিষয়ে যে তথ্য বা পরামর্শ আপনি পেয়েছিলেন সেগুলোকে কতটা কার্যকর করতে পেরেছিলেন?  Not at all ***একদমই না***……1  A little সামান্য…………….2  Well ভালো ………………..3  Very well ভালো**ভাবে** …..…4 | To what extent are you yourself (singular) able to access information that you feel is important for making informed decisions regarding [TOPIC]?  এই বিষয়ে নির্ণয় নিতে হলে যে তথ্য গুলো আপনার দরকারি বলে মনে হয় সেই ধরনের তথ্য পেতে আপনি নিজে (একক ভাবে ) কতটা সক্ষম?  Not at all ***একদমই না***……………1  A little সামান্য………………….2  Well ভালো ………………..3  Very well ভালো**ভাবে** …..…4 |
| --- | --- | --- | --- | --- | --- |
| **TOPIC** বিষয় | | **G3.19** | **G3.20** | **G3.21** | **G3.22** |
| **A** | Fish farming or marketing মাছ চাষ বা বিপণন |  |  |  |  |
| **B** | Agriculture farming or marketing কৃষিক্ষেত্র বা বিপণন |  |  |  |  |
| **C** | Weather (such as rain, temperature, etc.)  আবহাওয়া (যেমন বৃষ্টি, তাপমাত্রা ইত্যাদি) |  |  |  |  |
| **D** | Health related information (such as sanitation and hygiene; clean water; reproductive health)  স্বাস্থ্য সম্পর্কিত তথ্য (যেমন স্যানিটেশন এবং হাইজিন; পরিষ্কার জল; প্রজনন স্বাস্থ্য) |  |  |  |  |

MODULE G4: GROUP MEMBERSHIP INCLUDING INFLUENCE দল বা গোষ্ঠীর সদস্যপদ ও তার সঙ্গে যুক্ত ক্ষমতা, প্রতিপত্তি এবং প্রভাব

| Now I am going to ask you about groups in the community. These can be either formal or informal and customary groups. Community can be your village but also other groups that you share a common interest, education, religion, or social issues, that may go beyond your village.  এখন আমি আপনাকে সম্প্রদায়ের বিভিন্ন গ্রুপ (গোষ্ঠী) সম্পর্কে জিজ্ঞাসা করব। এগুলি হয় প্রচলিত বা আইনগত এবং অপ্রথাগত গ্রুপ হতে পারে। সম্প্রদায়টি আপনার গ্রাম হতে পারে। এমন গ্রুপও হতে পারে যেখানে আপনি একটি সাধারণ আগ্রহ, শিক্ষা, ধর্ম বা সামাজিক সমস্যাগুলি নিয়ে আলোচনা করেন যা আপনার গ্রামের বাইরের বিষয়। | | Is there a [GROUP] in your community?  আপনার সম্প্রদায়ের মধ্যে কোন [এই ধরনের গ্রুপ] আছে?  Yes **হ্যাঁ** 1  No **না** 2 **>> NEXT GROUP**  **পরবর্তী গ্রুপ**  Don’t know 97 **>> NEXT GROUP**  **পরবর্তী গ্রুপ** | Is this group composed of all men or all women or a mix of women and men members?  এই ধারণের গ্রুপ কি কেবল পুরুষ বা কেবল মহিলা সদস্য ডিয়ে তৈরি না মহিলা এবং পুরুষ দুজনেই সদস্য?  All Men সমস্ত পুরুষ…1  All Women সমস্ত মহিলা..2  Mixed Gender মিশ্র লিঙ্গ...3  Don’t know জানি না…97 | Are you an active member of this [GROUP], that is, involved in any of the group’s activities or decision making?  আপনি কি এই [গ্রুপ] এর একজন সক্রিয় সদস্য, অর্থাৎ, দলের কোনও কার্যক্রম বা সিদ্ধান্ত নেওয়ার সাথে জড়িত?  Yes **হ্যাঁ**.........1  No **না** ………2 **>> G4.06** | What role do you take in this [GROUP]?  আপনি এই [গ্রুপে] কী ভূমিকা নেন বা কাজ করেন?  Simple member  সাধারণ সদস্য.. 1  Leadership  নেতা / নেত্রী .......2 | To what extent do you feel like you can influence decisions in this GROUP?  ***আপনার মতে, এই গ্রুপের নির্ণয় গুলোরে আপনি কতটা প্রভাবিত করতে পারেন?***  Not at all একটুও নয়…1  Small অল্প...2  Medium মাঝারি...3  High অনেকটা …4 | To what extent does this group influence life in the community beyond the group activities?  এই গ্রুপ নিজের কাজ ছাড়া সমাজ জীবনকে কতটা প্রভাবিত করে?  Not at all একটুও নয়…1  Small অল্প...2  Medium মাঝারি...3  High অনেকটা …4  **>> NEXT GROUP**  **পরবর্তী গ্রুপ** |
| --- | --- | --- | --- | --- | --- | --- | --- |
| **GROUP CATEGORIES গ্রুপের ধরন** | | **G4.01** | **G4.02** | **G4.03** | **G4.04** | **G4.05** | **G4.06** |
| **A** | Agricultural / livestock/ fisheries producer’s group (including marketing groups) কৃষি / প্রাণিসম্পদ / মৎস্য উৎপাদকের গ্রুপ (বিপণন গ্রুপ সহ) |  |  |  |  |  |  |
| **B** | Community based resource management সম্প্রদায় ভিত্তিক রিসোর্স ম্যানেজমেন্ট |  |  |  |  |  |  |
| **C** | Fisherman committee মৎস্যজীবী কমিটি |  |  |  |  |  |  |
| **D** | Water users’ group জল ব্যবহারকারীদের গ্রুপ |  |  |  |  |  |  |
| **E** | Forest users’ group বন ব্যবহারকারীদের গ্রুপ |  |  |  |  |  |  |
| **F** | Credit or microfinance group (including SACCOs/merry-go-rounds/ VSLAs)  ক্রেডিট বা ক্ষুদ্রঋণ প্রদান গ্রুপ (ভিএসএলএ / স্যাকসিও / ম্যারি-গো-রাউন্ড) |  |  |  |  |  |  |
| **G** | Mutual help or insurance group (including burial societies) পারস্পরিক সহায়তা বা বীমা গ্রুপ (সমাধি সমিতি সহ) |  |  |  |  |  |  |
| **H** | Trade and business association  বাণিজ্য ও ব্যবসায়ী সমিতি |  |  |  |  |  |  |
| **I** | Civic groups (improving community) or charitable group (helping others) নাগরিক গোষ্ঠী (সম্প্রদায়ের উন্নতি) বা দাতব্য গোষ্ঠী (অন্যকে সহায়তা করা) |  |  |  |  |  |  |
| **J** | Local government স্থানীয় সরকার |  |  |  |  |  |  |
| **K** | Religious group ধর্মীয় দল |  |  |  |  |  |  |
| **L** | Other women’s group (only if it does not fit into one of the other categories)  অন্যান্য মহিলাদের গোষ্ঠী (কেবলমাত্র এটি অন্য বিভাগগুলির মধ্যে একটির সাথে ফিট করে না) |  |  |  |  |  |  |
| **M** | Social media groups (i.e. virtual group membership) সামাজিক মিডিয়া গোষ্ঠী (যেমন ভার্চুয়াল গ্রুপ সদস্যতা) |  |  |  |  |  |  |
| **N** | Other (specify) অন্যান্য, নির্দিষ্ট করুন |  |  |  |  |  |  |

MODULE G5: Physical Mobility **যাতায়াত**

| Now I’m going to ask you some questions about some places you may visit.  এখন আমি আপনার ভ্রমণ যোগ্য কিছু জায়গা সম্পর্কে জিজ্ঞাসা করতে যাচ্ছি। | | How often do you visit [PLACE]?  আপনি এই জায়গায় কত দিন অন্তর যান?  Everyday প্রতিদিন …….1  Every week at least once প্রতি সপ্তাহে কমপক্ষে একবার …….2  Every 2 weeks at least once প্রতি 2 সপ্তাহ অন্তত একবার……3  Every month at least once প্রতি মাসে অন্তত একবার……4  Less than once a month মাসে একবারেরও কম……5  Never কখনই না……6  Not specific, whenever required নির্দিষ্ট নয়/যখন দরকার....7  **IF RESPONDENT IS MALE** উত্তরদাতা যদি পুরুষ হন **🡪 NEXT PLACE** পরের জায়গা |
| --- | --- | --- |
|  |  |  |
| **PLACE জায়গা** | | **G5.01** |
| **A** | Urban center নগর বা শহর |  |
| **B** | Local market / haat / bazaar or trading place স্থানীয় বাজার / হাট / বাজার বা ব্যবসায়ের জায়গা |  |
| **C** | Family or relatives outside the village  **গ্রামের বাইরে পরিবার বা আত্মীয়স্বজন** |  |
| **D** | Family or relatives inside the village  গ্রামের ভিতরে পরিবার বা আত্মীয়স্বজন |  |
| **E** | Friend / neighbor’s house outside the village  গ্রামের বাইরে বন্ধু / প্রতিবেশীর বাড়ি |  |
| **F** | Friend/neighbor’s house inside the village  গ্রামের ভিতরে বন্ধু / প্রতিবেশীর বাড়ি |  |
| **G** | Hospital / clinic / doctor (seek health advice, receive treatment)  হাসপাতাল / ক্লিনিক / ডাক্তার (স্বাস্থ্যের পরামর্শ নিতে, চিকিৎসা গ্রহণ করতে) |  |
| **H** | Public village gathering / village meeting/training for NGO or programs সরকারী গ্রাম সমাবেশ / গ্রাম সভা / এনজিও বা প্রোগ্রামগুলির প্রশিক্ষণ নিতে |  |
| **I** | Public religious place (pagoda, monastery, church, mosque)  পাবলিক ধর্মীয় স্থান (মসজিদ,মঠ, গির্জা প্যাগোডা,) |  |

## MODULE G6: INTRAHOUSEHOLD RELATIONSHIP *পরিবারের মধ্যে সম্পর্ক*

| Now I’d like to ask you some questions about how you feel about some of other people in your household or family group and how you think they feel about you.  এখন আমি আপনার পরিবারের বা পারিবারিক গোষ্ঠীর অন্যান্য ব্যক্তির সাথে আপনার সম্পর্ক সম্বন্ধে কিছু প্রশ্ন জিজ্ঞাসা করতে চাই, যাঁদের সম্পর্কে আপনি কিভাবেন এবং আপনার সম্পর্কে তাঁরা কি অনুভব করেন বলে আপনার ধারনা।  ***[A or B are meant to be whoever is the other WEFI decision maker;*** A অথবা B এই সমীক্ষায় পরিবারের অন্য নির্ণায়ক সদস্য  **ENTER MEMBER ID FOR EACH RELATION**  **প্রতিটি সম্পর্কের জন্য সদস্য আইডি প্রবেশ করান**  ***[If not applicable, write 98 and skip to the next row]***  **প্রযোজ্য না হলে 98 লিখুন এবং পরবর্তী সারিতে যান** | | Who Is the other respondent to this survey within this household?  এই সমীক্ষার অন্য উত্তরদাতা ব্যাক্তির সাথে আপনার সম্পর্ক কী? | Do you [NAME] respect your [RELATION]?  আপনি কি এই ব্যাক্তিকে সম্মান করেন?  Most of the time  বেশিরভাগ সময়…..1  Sometimes  কখনও কখনও….2  Rarely কদাচিৎ….3  Never কখনই না……4 | Does your RELATION respect you?  এই ব্যাক্তি কি আপনাকে শ্রদ্ধা করেন?  Most of the time  বেশিরভাগ সময়…..1  Sometimes  কখনও কখনও….2  Rarely কদাচিৎ….3  Never কখনই না……4 | Do you trust your [RELATION] to do things that are in your best interest?  আপনি কি বিশ্বাস করেন যে এই ব্যাক্তি যা করেন তা আপনার ভালর জন্যেই?  Most of the time  বেশিরভাগ সময়…..1  Sometimes  কখনও কখনও….2  Rarely কদাচিৎ….3  Never কখনই না……4 | When you disagree with your [RELATION], do you feel comfortable telling him/her that you disagree?  আপনি যদি কখন এই ব্যাক্তির সাথে একমত না হন , আপনি কি তাঁকে সেটা জানাতে স্বাচ্ছন্দ্য বোধ করেন?  Most of the time  বেশিরভাগ সময়…..1  Sometimes  কখনও কখনও….2  Rarely কদাচিৎ….3  Never কখনই না……4 | Is there a co-wife within your household?  আপনার সংসারে কি কোন সতীন আছে? |
| --- | --- | --- | --- | --- | --- | --- | --- |
| **RELATION সম্পর্ক** | | **G6.01** | **G6.02** | **G6.03** | **G6.04** | **G6.05** | **G6.06** |
| **A** | Partner / Spouse সঙ্গী / স্ত্রী অথবা স্বামী | Yes **হ্যাঁ** …..1  No না…….2 |  |  |  |  |  |
| **B** | Other relation within household (Specify) পরিবারের মধ্যে অন্য সম্পর্ক (বিবরণ দিন) |  |  |  |  |  |  |

## MODULE G7 (A): AUTONOMY IN INCOME *রোজগার করা ও সেই টাকা ব্যবহার করার স্বাধীনতা*

| Now I’d like to ask you some questions about your income.  **এখন আমি আপনাকে আপনার রোজগার অথবা আয়ের বিষয়ে কিছু প্রশ্ন জিজ্ঞাসা করতে চাই।** | | **Code 11** |
| --- | --- | --- |
|  |  | Fish **মাছ** ……………………….1  Crop শস্য …………,,,,,,,,,,,,...2  Livestock পশুসম্পদ ………...3  Business ব্যবসা………………...4  Other অন্যান্য………...............97  None (do not earn an income of one’s own) কিছুই নয় (কারও নিজস্ব উপার্জন করবেন না)…………..0 |
| **G7.01** Here are 10 beans. The beans together represent all the resources your household needs, such as food, clothes, schooling and more. From what you yourself earn (your income from all sources), how many beans represent your contribution to meeting these needs?  **মনে করুন,** 10টি **মটরশুটি** আছে। **মটরশুটিগুলি একসাথে আপনার পরিবারের প্রয়োজনীয় সমস্ত সংস্থান যেমন খাদ্য, বস্ত্র, স্কুল পড়াশোনা ইত্যাদির নিদর্শন। এই সবকিছুর খরচ মেটাতে আপনার অবদান, আপনার নিজের রোজগার (সমস্ত উ**ৎস **থেকে আপনার রোজগার) কে কতগুলি মটরশুঁটি দিয়ে চিহ্নত করবেন?** | \|  \|  \| \| --- \| --- \|   **FILL WITH NUMBER 0 TO 10.**  **0 থেকে 10 নম্বর দিয়ে পূরণ করুন।**  **0 🡪 G7.04** |  |
| **G7.02** What is the main source of your income that you earn yourself? (individual income)  **আপনার নিজের রোজগারের প্রধান উ**ৎস **কী? (আপনার একার রোজগার)**  **Code 11** *কোড* **11** | \|  \|  \| \| --- \| --- \| |  |
| **G7.03** What are the main categories you spend the money that you earn yourself?  **আপনি নিজে যা রোজগার করেন, কোন কোন জায়গায় প্রধানত খরচ করেন?**  **Code 12** *কোড* **12** | \|  \| \| --- \|  \|  \| 1st spending source  প্রথম খরচের জায়গা \| \| --- \| --- \| \|  \| 2nd spending source  দ্বিতীয় খরচের জায়গা \| \|  \| 3rd spending source  তৃতীয় খরচের জায়গা \|   **ENTER UP TO THREE CODES**  *তিনটি পর্যন্ত কোড ব্যবহার করা যাবে* | **Code 12** |
|  |  | Food খাদ্য 1  Other household necessities পরিবারের অন্যান্য প্রয়োজনীয়তা 2  Rent or mortgage ভাড়া বা বন্ধক 3  Utilities (water, electricity, cooking fuel) উপযোগ (জল, বিদ্যুৎ, রান্নার জ্বালানী) 4  Communication (phone, airtime, internet) যোগাযোগ (ফোন, এয়ারটাইম, ইন্টারনেট)……………………………………………………………………5  Transportation (fuel, maintenance, taxi) পরিবহন (জ্বালানী, রক্ষণাবেক্ষণ, ট্যাক্সি)…………………………………………………………………………6  Education (school fees, books, supplies) শিক্ষা (স্কুল ফি, বই, সরবরাহ)  7  Health care (visits, medicine, insurance স্বাস্থ্যসেবা (পরিদর্শন, ওষুধ, বীমা)  8  Clothes and other personal items (shoes) কাপড় এবং অন্যান্য ব্যক্তিগত আইটেম (জুতা) 9  Business related expenses (farm, aquaculture, etc.) ব্যবসায় সম্পর্কিত ব্যয় (খামার, জলজ পালন ইত্যাদি……………………………………………..10  Taxes কর 11  Leisure/hobbies (bar, concert, festival, sports, alcohol/cigarettes) অবসর / শখ (বার, কনসার্ট, উৎসব, ক্রীড়া, অ্যালকোহল / সিগারেট) 12 |

| Now I am going to read you some stories about different farmers and their situations regarding different agricultural activities. This question format is different from the rest so take your time in answering. For each I will then ask you how much you are like or not like each of these people. We would like to know if you are completely different from them, similar to them, or somewhere in between. There are no right or wrong answers to these questions.  এখন আমি আপনাকে বিভিন্ন কৃষক এবং বিভিন্ন কৃষি কার্যক্রম সম্পর্কিত তাদের পরিস্থিতি সম্পর্কে কিছু গল্প পড়ে শোনাচ্ছি। এই প্রশ্নের ধরন অন্য প্রশ্নের থেকে আলাদা তাই ভেবে-চিনতে সময় নিয়ে উত্তর দিন। প্রত্যেক ক্ষেত্রে আমি আপনাকে জিজ্ঞাসা করব, যে আপনি কতখানি এই লোকগুলির মতো । আমরা জানতে চাই আপনি কি একেবারে তাঁদের মতো, না সম্পূর্ণ আলাদা, নাকি মাঝামাঝি কিছু? এই প্রশ্নের কোন সঠিক বা ভুল উত্তর নেই।  **READ ALOUD EACH STORY, SUBSEQUENT QUESTIONS, AND RESPONSE CODES.**  প্রতিটি গল্প, পরবর্তী প্রশ্ন এবং প্রতিক্রিয়া কোড পড়ুন।  **NAMES SHOULD BE ADOPTED TO LOCAL CONTEXT AND BE MALE/FEMALE DEPENDINGO N THE GENDER OF THE RESPONDENT (SHOULD NOT BE THE SAME AS THE RESPONDENT’S NAME).**  **না**মগুলি পুরুষ / মহিলা নির্ভর করে স্থানীয় হওয়া উচিত (উত্তরদাতার নাম হওয়া উচিত নয়।)  **THE ORDER OF STORIES A-C SHOULD BE RANDOMIZED. গল্পের ক্রম A থেকে C পর পর না বলে, মিলিয়ে মিশিয়ে বলা উচিত।** | | Are you like this person?  আপনি কি এই ব্যক্তির মত?  **SELECT ONE**  যেকোনো একটি চিহ্নিত করুন  Yes হ্যাঁ = 1  No না = 2 **🡪** **G7.05** |
| --- | --- | --- |
|  | **STORY** | **G7.04** |
| **A** | *“[PERSON’S NAME] uses her income how her family or community tell her she must use it. She does what they tell her to do.”*  *এই ব্যাক্তি* ( *নাম ) তাঁর রোজগার, যে ভাবে তাঁর পরিবার বা সম্প্রদায় তাঁকে ব্যবহার করতে বলেন, ঠিক সেই ভাবেই ব্যবহার করেন। তাঁরা তাঁকে যা করতে বলেন তিনি তাই করেন।* | **A** |
| **B** | *“[PERSON’S NAME] uses her income in the way that her family or community expect. She wants them to approve of her.”*  *এই ব্যাক্তি* ( *নাম ) তাঁর রোজগার, পরিবার বা সম্প্রদায় যা প্রত্যাশা করে সেইভাবে ব্যবহার করে। তিনি চান যে তাঁর পরিবার বা সম্প্রদায় তাঁকে অনুমোদন করুক।* | **B** |
| **C** | *“[PERSON’S NAME] chooses to use her income how she personally wants to, and thinks is best for herself and her family. She values using her income in this way. If she changed her mind, she could act differently.”*  *এই ব্যাক্তি* (*নাম ) নিজে যেমন ভাবে চান তেমন ভাবেই তাঁর রোজগারের ব্যবহার বেছে নেন, বিশেষত*  *যা তাঁর নিজের এবং তাঁর পরিবারের পক্ষে সবচেয়ে ভাল বলে মনে করেন। তিনি নিজের রোজগারকে এইভাবেই ব্যবহার করতে পছন্দ করেন। তিনি যদি মত পরিবর্তন করেন তবে তিনি সেই পরিবর্তিত মত আমল করতে পারতেন।* | **C** |

## MODULE G7 (B): Self Efficacy *স্ব-কার্যকারিতা স্কেল* (Re-adapted from Bandura 2006) (বান্দুরা 2006 থেকে পুনরায় রূপান্তরিত)

This questionnaire is designed to help us get a better understanding of the kinds of things that create difficulties for people like you in their daily life. Please rate how certain you are that you can do the things I ask on a scale from 1 – 5. Think about how each statement relates to your life, and then tell me how certain you are that you can do the things I ask on a scale from 1 to 5 with 1 meaning that you cannot do it at all, 3 meaning you can sometimes do it and 5 that you can certainly do it.

**এই প্রশ্নাবলি আপনাদের মত মানুষদের দৈনন্দিন জীবনে যে সমস্ত অসুবিধা হয়, সেই সব অসুবিধা গুলিকে আমাদের ভাল করে বুঝতে সাহায্য করবে। প্রতিটি বিবৃতি আপনার জীবনের সাথে কীভাবে সম্পর্কিত তা ভেবে দেখুন এবং তারপরে আমাকে বলুন যে আপনি 1 থেকে 5 স্কেলের বিবৃতিতে আপনি কত খানি সক্ষম? ১ মানে আপনি একেবারেই পারবেননা, ৩ মানে কখন কখন করতে পারেন এবং ৫ মানে অবশ্যই করেতে পারেন।**

| **G7.05** | **Question প্রশ্ন**  Please express to what extent you agree with the FOLLOWING statements  **আপনি নিম্নলিখিত বিবৃতিগুলির সাথে কতখানি একমত** | **Response উত্তর** | **Response code উত্তর কোড** |
| --- | --- | --- | --- |
| **A** | How well can you influence the decisions that are made in the household? **আপনার গৃহস্থের সিদ্ধান্তগুলিকে আপনি কতটা প্রভাবিত করতে পারেন?** |  | Cannot at all একেবারেই পারিনা 1  Cannot usually do সাধারণত করতে পারিনা......2  Can sometimes do কখন-সখন করতে পারি........3  Can usually do সাধারণত করতে পারি.......4  Can certainly do অবশ্যই করেতে পারি.......5 |
| **B** | How well can you express your views freely on important household matters? পরিবারের **গুরুত্বপূর্ণ বিষয়গুলিতে আপনি অবাধে আপনার মতামত প্রকাশ করতে পারেন?** |  |  |
| **C** | How well can you get the money you need for the household expenses? পরিবারের খরচ গুলোর জন্য প্রয়োজনীয় অর্থ সহজে পেয়ে থাকেন? |  |  |
| **D** | How well can you get the equipment/material you need for your activities? আপনার কাজের জন্য প্রয়োজনীয় সরঞ্জাম / জিনিসপত্র সহজে পেয়ে থাকেন? |  |  |
| **E** | How well can you express your views freely on important community matters? জরুরি সামাজিক বিষয়ে স্বাধীন ভাবে আপনার মতামত প্রকাশ করতে পারেন? |  |  |
| **F** | How well can you remain confident during difficult times? কঠিন (অসুবিধাজনক অবস্থা) সময়ে আপনার আত্মবিশ্বাস বজায় রাখতে পারেন? |  |  |
| **G** | How well can you bounce back quickly from adverse experiences? কোন খারাপ অভিজ্ঞতা থেকে তাড়াতাড়ি নিজেকে সামলে নেন? |  |  |
| **H** | How well can you get family members to share household responsibilities? আপনি সহজেই পরিবারের সদস্যদের মধ্যে বিভিন্ন দায়িত্ব ভাগ করে দিতে পারেন? |  |  |
| **I** | How well can you achieve most of the goals that you have set for myself? আপনি সহজেই নিজের জন্য স্থির করা বেশিরভাগ লক্ষ্য সফলভাবে অর্জন করেন? |  |  |

##

## MODULE G8: Attitude toward domestic violenceপারিবারিক সহিংসতার প্রতি মনোভাব

Now I am going to ask you your opinion on the following issues. Please keep in mind that I am not asking you about your personal experience or whether the following scenarios have happened to you. I would only like to know whether you think the following issue is acceptable.

**এখন আমি কিছু বিষয়ে আপনার মতামত জিজ্ঞাসা করব। দয়া করে মনে রাখবেন যে আমি আপনাকে আপনার ব্যক্তিগত অভিজ্ঞতা বা বিভিন্ন পরিস্থিতিতে আপনার সাথে ঘটেছে কিনা তা সম্পর্কে জিজ্ঞাসা করছি না। আপনি কেবল নিম্নলিখিত বিষয়টি আপনার জন্য গ্রহণযোগ্য কিনা, সেটা জানতে চাই।**

| **G8.01** | **Question** | **Response code**  Yes **হ্যাঁ** …………………..1  No না ……...........................2  Don’t’ know জানি না ………...97 |
| --- | --- | --- |
| **A** | In your opinion, is it okay for a husband to hit or beat his wife if she goes out without telling him? **আপনার মতে, একজন স্ত্রী যদি তার স্বামীকে কিছু না বলে বাইরে চলে যায় তবে তাকে তার স্বামীর মারধর করা কি ঠিক?** |  |
| **B** | In your opinion, is it okay for a husband to hit or beat his wife if she neglects the children? **আপনার মতে, একজন স্ত্রী যদি বাচ্চাদের অবহেলা করে তবে তাকে তার স্বামীর মারধর করা কি ঠিক?** |  |
| **C** | In your opinion, is it okay for a husband to hit or beat his wife if she argues with him? **আপনার মতে, একজন স্ত্রী যদি তার স্বামীর সাথে তর্ক করে তবে তাকে তার স্বামীর মারধর করা কি ঠিক?** |  |
| **D** | In your opinion, is it okay for a husband to hit or beat his wife if she refuses to have sex with him? **আপনার মতে, একজন স্ত্রী যদি তার স্বামীর সাথে সহবাস করতে অস্বীকার করে তবে তাকে তার স্বামীর মারধর করা কি ঠিক?** |  |
| **E** | In your opinion, is it okay for a husband to hit or beat his wife if she burns the food? **আপনার মতে, একজন স্ত্রী যদি রান্নার সময় খাবার পুড়িয়ে ফেলে তবে তাকে তার স্বামীর মারধর করা কি ঠিক?** |  |
| **F** | In your opinion, is it okay for a husband to hit or beat his wife if she decides how to use income (i.e. spends money) without his permission? **আপনার মতে, একজন স্ত্রী যদি তার স্বামীর অনুমতি ছাড়া পরিবারের রোজগার কি ভাবে খরচ করা হবে সে বিষয়ে সিদ্ধান্ত নেয় তবে তাকে তার স্বামীর মারধর করা কি ঠিক?** |  |
| **G** | In your opinion, is it okay for a husband to hit or beat his wife if she does not support her husband in his work? **আপনার মতে, একজন স্ত্রী যদি তার স্বামীকে মাছ ধরা / কৃষি কাজে সাহায্য না করেন তবে তাকে তার স্বামীর মারধর করা কি ঠিক?** |  |
| **H** | In your opinion, is it okay for a husband to hit or beat his wife if she attends trainings or meetings without telling him? **আপনার মতে, একজন স্ত্রী যদি তার স্বামীকে না বলে কোন প্রশিক্ষণ অথবা কোন সভায় অংশগ্রহণ করে তবে তাকে তার স্বামীর মারধর করা কি ঠিক?** |  |

## MODULE G9: GENDER ATTITUDES লৈঙ্গিক আচরণ

The purpose of this section is to get information about existing gender attitudes relevant within the fish value chain. Enumerator: Please explain that this is about HER OPINION about these statements, not about what is happening in practice.

এই বিভাগের উদ্দেশ্য হ'ল **ফিশ ভ্যালু চেইনের** মধ্যে প্রাসঙ্গিক লৈঙ্গিক আচরণ এবং মনোভাব সম্পর্কিত তথ্য পাওয়া। সার্ভেয়ররা পরিষ্কার ভাবে উত্তরদাতাদের বোঝাবেন যে এই বিবৃতিগুলি সম্পর্কে তার (উত্তরদাতার) মতামত জরুরি, এমনটা বাস্তবে না ঘটে থাকতে পারে।

| **G9.01** | **Statement বিবৃতি** | **READ ALOUD THESE OPTIONS :**  Strongly agree অত্যন্ত সম্মত …….1  Agree সম্মত.................2  Neither agree nor Disagree সম্মত বা অসম্মত কোনটাই নয় .....3  Disagree অসম্মত ............... 4  Strongly disagree অত্যন্ত অসম্মত ...…5 |
| --- | --- | --- |
| **A** | Women should leave fish farming to the men especially if there is an able man in the household to do it. মহিলাদের মাছ চাষের কাজ পুরুষদের কাছে ছেড়ে দেওয়া উচিৎ বিশেষত যদি পরিবারের কোনও সক্ষম পুরুষ তা করতে পারে। |  |
| **B** | A man is less of a man if his wife is involved in fish farming. একজন স্ত্রী যদি মাছ চাষের সাথে জড়িত থাকলে তবে সেই পরিবারের পুরুষের পৌরুষ অন্য পুরুষদের চেয়ে কম হয়ে যায়। |  |
| **C** | If a woman travels to suppliers of inputs or fish buyers or markets she deserves to be harassed. যদি কোনও মহিলা ইনপুট বা মাছ -ক্রেতা বা বাজারের সরবরাহকারীদের কাছে নিয়ে যায় তবে তিনি হয়রানির শিকার হন। |  |
| **D** | Women should not own fish ponds or ghers. মহিলাদের মাছের পুকুর বা ঘেরের/ভেরির মালিক হওয়া উচিত নয় |  |
| **E** | Women should not own fishing nets, and other equipment used for fish farming. মহিলাদের মাছ ধরার জাল এবং মাছ চাষের জন্য ব্যবহৃত অন্যান্য সরঞ্জামের মালিক হওয়া উচিত নয় |  |
| **F** | Women should primarily be the ones who clean and process fish. মহিলাদের প্রধানতঃ মাছ পরিষ্কার এবং প্রক্রিয়াজাত করার কাজে যুক্ত থাকা উচিত |  |
| **G** | Men should be responsible for marketing fish, not women. মাছ বিপণনের জন্য পুরুষদের দায়িত্বশীল হওয়া উচিত, এটা মহিলাদের কাজ নয়। |  |
| **H** | Men should primarily be the ones who transport fish to a market. পুরুষদেরই প্রধানত বাজারে মাছ পরিবহন করা উচিত। |  |
| **I** | Men should primarily be the ones who control the earnings obtained from the sale of fish. মাছ বিক্রয় থেকে পাওয়া টাকার (রোজগার) উপর প্রধানত পুরুষদেরই নিয়ন্ত্রণ থাকা উচিত। |  |
| **J** | Women should primarily be the ones who prepare meals (including fish) for the family. মহিলাদেরই প্রধানত পরিবারের লোকদের জন্য খাবার (মাছ রান্না সহ) প্রস্তুত করার কাজ উচিত। |  |
| **K** | Men should mostly be the ones who belong to aquaculture or fisheries clubs, organizations, or associations, not women. পুরুষদেরই বেশিরভাগ ক্ষেত্রে জলজ প্রাণী চাষ বা ফিশারি ক্লাব, সংস্থা বা সংঘের সদস্য হওয়া উচিত, মহিলারা এতে না থাকাই ভালো। |  |
| **L** | It brings benefit to the family to have women participate in training activities, such as aquaculture trainings. মাছ/জলজ প্রাণী চাষ প্রশিক্ষণের মতো প্রশিক্ষণ কার্যক্রমে মহিলাদের অংশগ্রহণ করা পরিবারের পক্ষে লাভজনক। |  |

**MODULE G10: CONSUMPTION AND CONSUMPTION HABITS** খাবার এবং খাওয়া-দাওয়ার অভ্যাস

*Enumerator: Ask this section to the female head/spouse or member who has the most knowledge on food preparation. সার্ভেয়ররা* এই প্রশ্নগুলো গৃহিণী বা পরিবারের সেই সদস্য কে করবেন যিনি খাবার তৈরি/রান্না করা সম্পর্কে সব থেকে বেশী জানেন।

| **Question** | | **Code** | **Response** |
| --- | --- | --- | --- |
| **G10.01** | In the past 4 weeks was there ever no food to eat of any kind in your house because of lack of resources to get food? গত 4 সপ্তাহের ভিতর কখন কি এমন অবস্থা হয়েছে যে খাবার জিনিস /রানার ব্যবস্থা না থাকার জন্য পরিবারে কোন প্রকারের খাবার ছিল না? | 1 = Yes হ্যাঁ  2 = No না (if No >>G10.03 যদি না >>**G10.03**) |  |
| **G10.02** | How often did this happen in the past 4 weeks? গত 4 সপ্তাহের ভিতর এরকম কতবার হয়েছে? | 1 = Rarely কদাচিৎ (1-2 times 1-2বার)  2 = Sometimes কখনও কখনও (3-10 times 3-10বার)  3 = Often অধিকাংশ সময় (> 10 times 10 বারের বেশী) |  |
|  |  |  |  |
|  |  |  |  |
| **G10.03** | In the past 4 weeks did you or any household member go to sleep at night hungry because there was not enough food? গত 4 সপ্তাহের ভিতর কখন কি আপনাকে অথবা আপনার পরিবারের কোন সদস্যকে যথেষ্ট খাবারের অভাবে খিদে পেটে ঘুমাতে হয়েছে? | 1 = Yes হ্যাঁ  2 = No না (if No >>G10.05 যদি না >>**G10.05**) |  |
|  |  |  |  |
|  |  |  |  |
| **G10.04** | How often did this happen in the past 4 weeks? গত 4 সপ্তাহের ভিতর এরকম কতবার হয়েছে? | 1 = Rarely কদাচিৎ (1-2 times 1-2বার)  2 = Sometimes কখনও কখনও (3-10 times 3-10বার)  3 = Often অধিকাংশ সময় (> 10 times 10 বারের বেশী) |  |
|  |  |  |  |
|  |  |  |  |
| **G10.05** | In the past 4 weeks did you or any household member go a whole day and night without eating anything at all because there was not enough food? গত 4 সপ্তাহের ভিতর কখন কি আপনাকে অথবা আপনার পরিবারের কোন সদস্যকে যথেষ্ট খাবারের অভাবে সারাদিন এবং সারারাত খিদে নিয়ে থাকতে হয়েছে? | 1 = Yes হ্যাঁ  2 = No না (if No >> G11 module যদি না >> G11 মডিউল) |  |
|  |  |  |  |
|  |  |  |  |
|  |  |  |  |
| **G10.06** | How often did this happen in the past 4 weeks? গত 4 সপ্তাহের ভিতর এরকম কতবার হয়েছে? | 1 = Rarely কদাচিৎ (1-2 times 1-2বার)  2 = Sometimes কখনও কখনও (3-10 times 3-10বার)  3 = Often অধিকাংশ সময় (> 10 times 10 বারের বেশী) |  |
|  |  |  |  |
|  |  |  |  |
|  |  |  |  |

**MODULE G11. NUTRITION AND HEALTH** পুষ্টি এবং স্বাস্থ্য

| Now I’d like to ask you some questions on *making decisions about your health and nutrition.*  এবার আপনাকে কিছু স্বাস্থ্য এবং খাদ্যের পুষ্টির বিষয়ে প্রশ্ন করব। এই প্রশ্ন গুলো ওই বিষয়ে আপনার নির্ণয় বা সিধান্ত নেওয়া সঙ্গে জড়িত।  [Note for survey adaptation: Based on specific needs, projects may wish to provide more detailed response categories for **other non-household family members** (response code 6) listed for question GX.01 and GX.04. Example additions might include, RESPONDENT’S MOTHER (if outside household)…81; RESPONDENT’S MOTHER-IN-LAW (if outside household)…82; A SENIOR CO-WIFE (if outside household)…83; ABSENT HUSBAND…84. These response codes should be in the 80s.] | | Who is normally involved in making decisions about [ACTIVITY]?  সাধারণত এই বিষয়ে নির্ণয় কে বা কাঁরা নিয়ে থাকেন?  **ENTER UP TO THREE (3) CODES সর্বাধিক তিনটি কোড বাছতে পারেন**  **IF RESPONSE IS MEMBER ID (SELF) ONLY** 🡪 ***GX.03 উত্তর যদি নিজে হয়, GX.03 তে যান***  **CODES:**  Self নিজে …………1  Partner/Spouse in HH পরিবারে বসবাসকারী সঙ্গী/স্বামী/স্ত্রী …………2  Partner/Spouse outside HH পরিবারের থেকে দূরে থাকা সঙ্গী/স্বামী/স্ত্রী …………3  Other HH member পরিবারের অন্য সদস্য…………………4  NON-HH, NON-FAMILY MEMBER পরিবারের বাইরে থাকা ব্যক্তি যে পরিবারের সদস্য নন 5  Non-hh, FAMILY MEMBER পরিবারের বাইরে থাকা ব্যক্তি যে পরিবারের সদস্য 6  NOT APPLICABLE প্রযোজ্য নয়/ সিদ্ধান্ত নেওয়া হয়নি 98 >> NEXT Row পরের বিষয় | | | To what extent do you participate in decisions regarding [ACTIVITY]?  এই বিষয়ে **সিদ্ধান্ত** নেয়ায় আপনি কতটা অংশগ্রহণ করেন?  **CIRCLE ONE**  যেকোন একটিকে চিহ্নিত করুন | How confident do you feel to make decisions about [ACTIVITY]?  এই বিষয়ে **সিদ্ধান্ত** নিতে আপনি কতটা অসংশয়/আত্মবিশ্বাসী বোধ করেন?  **CIRCLE ONE**  যেকোন একটিকে চিহ্নিত করুন | When decisions are made regarding [ACTIVITY], who would you prefer made the decision?  আপনার মতে কার বা কাদের এই বিষয়ে **সিদ্ধান্ত নেওয়া উচিত**?  **ENTER UP TO THREE (3) CODES সর্বাধিক তিনটি কোড বাছতে পারেন**  **CODES:**  Self নিজে …………1  Partner/Spouse in HH পরিবারে বসবাসকারী সঙ্গী/স্বামী/স্ত্রী …………2  Partner/Spouse outside HH পরিবারের থেকে দূরে থাকা সঙ্গী/স্বামী/স্ত্রী ……3  Other HH member পরিবারের অন্য সদস্য…………………4  NON-HH, NON-FAMILY MEMBER পরিবারের বাইরে থাকা ব্যক্তি যে পরিবারের সদস্য নন 5  Non-hh, FAMILY MEMBER পরিবারের বাইরে থাকা ব্যক্তি যে পরিবারের সদস্য 6  NOT APPLICABLE প্রযোজ্য নয়/ সিদ্ধান্ত নেওয়া হয়নি 98 >> NEXT Row পরের বিষয় | | | |
| --- | --- | --- | --- | --- | --- | --- | --- | --- | --- | --- |
| **WOMAN’S HEALTH AND NUTRITION** | | **G11.01** | | | **G11.02** | **G11.03** | **G11.04** | | | |
|  |  | **ID #1** | **ID #2** | **ID #3** |  |  | **ID #1** | | **ID #2** | **ID #3** |
| **A** | Whether or not you consult a doctor or go to a clinic when you are ill? আপনার শরীর খারাপ হলে আপনি ডাক্তারের পরামর্শ নেবেন কিনা বা কোন স্বাস্থ্য কেন্দ্রের যাবেন কিনা? |  |  |  | Not at all একটুও নয়…1  Small extent অল্প পরিমাণ...2  Medium extent মাঝারি পরিমাণ...3  To a high extent উচ্চ মাত্রায় …4 | Not at all একটুও নয় …1  Somewhat কিছুটা 2  Very confident একেবারে অসংশয়/আত্মবিশ্বাসী 3 |  | |  |  |
| **B** | How much you can rest when you are ill? আপনার শরীর খারাপ হলে আপনি কত খানি বিশ্রাম নিতে পারবেন? |  |  |  | Not at all একটুও নয়…1  Small extent অল্প পরিমাণ...2  Medium extent মাঝারি পরিমাণ...3  To a high extent উচ্চ মাত্রায় …4 | Not at all একটুও নয় …1  Somewhat কিছুটা 2  Very confident একেবারে অসংশয়/আত্মবিশ্বাসী 3 |  | |  |  |
| **C** | What foods to prepare every day? প্রতিদিন কি খাবার তৈরি করবেন? |  |  |  | Not at all একটুও নয়…1  Small extent অল্প পরিমাণ...2  Medium extent মাঝারি পরিমাণ...3  To a high extent উচ্চ মাত্রায় …4 | Not at all একটুও নয় …1  Somewhat কিছুটা 2  Very confident একেবারে অসংশয়/আত্মবিশ্বাসী 3 |  | |  |  |
| **D** | What foods (available in the house) you can eat? কি-কি খাবার (যা আপনার বাড়িতে উপলব্ধ) আপনি খেতে পারেন? |  |  |  | Not at all একটুও নয়…1  Small extent অল্প পরিমাণ...2  Medium extent মাঝারি পরিমাণ...3  To a high extent উচ্চ মাত্রায় …4 | Not at all একটুও নয় …1  Somewhat কিছুটা 2  Very confident একেবারে অসংশয়/আত্মবিশ্বাসী 3 |  | |  |  |
| **G11.05** Have you been pregnant or given birth within the past 4 years*? (Includes currently pregnant women) গত 4 বছরে কি আপনি কখন অন্তস্বত্বা হয়েছেন অথবা সন্তান প্রসব করেছেন? (বর্তমান অবস্থায় অন্তস্বত্বা মহিলাদেরও নেওয়া হবে)  [Note for survey adaptation: The timeframe highlighted for **G11.05** should be altered to reflect the program implementation period, such that women are only being asked this question if they were pregnant after the start of program implementation. Ideally, this same timeframe should be used at all surveys.] | | | | | | | | YES হ্যাঁ……..…….. 1  NO না……...……… 2 🡪 ***G11.06*** | | |
| **E** | Whether you consulted a doctor or went to a clinic *during your current or most recent pregnancy*? বর্তমান বা গত অন্তস্বত্বা অবস্থায় আপনি কি ডাক্তারের থেকে পরামর্শ নিয়েছেন অথবা কোন স্বাস্থ্য কেন্দ্রে গেছেন? |  |  |  | Not at all একটুও নয়…1  Small extent অল্প পরিমাণ...2  Medium extent মাঝারি পরিমাণ...3  To a high extent উচ্চ মাত্রায় …4 | Not at all একটুও নয় …1  Somewhat কিছুটা 2  Very confident একেবারে অসংশয়/আত্মবিশ্বাসী 3 |  | |  |  |
| **F** | How much you worked *during your current or most recent pregnancy*? বর্তমান বা গত অন্তস্বত্বা অবস্থায় আপনি কতটা কাজ করেছেন? |  |  |  | Not at all একটুও নয়…1  Small extent অল্প পরিমাণ...2  Medium extent মাঝারি পরিমাণ...3  To a high extent উচ্চ মাত্রায় …4 | Not at all একটুও নয় …1  Somewhat কিছুটা 2  Very confident একেবারে অসংশয়/আত্মবিশ্বাসী 3 |  | |  |  |
| **G** | How much you could rest *during your current or most recent pregnancy*? বর্তমান বা গত অন্তস্বত্বা অবস্থায় আপনি কতটা বিশ্রাম নিতে পেরেছিলেন অথবা পারছেন? |  |  |  | Not at all একটুও নয়…1  Small extent অল্প পরিমাণ...2  Medium extent মাঝারি পরিমাণ...3  To a high extent উচ্চ মাত্রায় …4 | Not at all একটুও নয় …1  Somewhat কিছুটা 2  Very confident একেবারে অসংশয়/আত্মবিশ্বাসী 3 |  | |  |  |
| **H** | Whether you could eat eggs *during your current or most recent pregnancy*? বর্তমান বা গত অন্তস্বত্বা অবস্থায় আপনি কি ডিম খেতে পেয়েছিলেন অথবা পারছেন/পাচ্ছেন? |  |  |  | Not at all একটুও নয়…1  Small extent অল্প পরিমাণ...2  Medium extent মাঝারি পরিমাণ...3  To a high extent উচ্চ মাত্রায় …4 | Not at all একটুও নয় …1  Somewhat কিছুটা 2  Very confident একেবারে অসংশয়/আত্মবিশ্বাসী 3 |  | |  |  |
| **I** | Whether you could consume milk or milk products *during your current or most recent pregnancy*? বর্তমান বা গত অন্তস্বত্বা অবস্থায় আপনি কতটা দুধ এবং দুধ জাত খাদ্য খেতে পেরেছিলেন/পেয়েছিলেন অথবা পারছেন/পাচ্ছেন? |  |  |  | Not at all একটুও নয়…1  Small extent অল্প পরিমাণ...2  Medium extent মাঝারি পরিমাণ...3  To a high extent উচ্চ মাত্রায় …4 | Not at all একটুও নয় …1  Somewhat কিছুটা 2  Very confident একেবারে অসংশয়/আত্মবিশ্বাসী 3 |  | |  |  |
| **J** | Whether you could eat meat, poultry or fish *during your current or most recent pregnancy*? অন্তস্বত্বা অবস্থায় আপনি কি মাছ- মাংস (সব রকম)খেতে পেরেছিলেন/পেয়েছিলেন অথবা পারছেন/পাচ্ছেন? |  |  |  | Not at all একটুও নয়…1  Small extent অল্প পরিমাণ...2  Medium extent মাঝারি পরিমাণ...3  To a high extent উচ্চ মাত্রায় …4 | Not at all একটুও নয় …1  Somewhat কিছুটা 2  Very confident একেবারে অসংশয়/আত্মবিশ্বাসী 3 |  | |  |  |
| **K** | How much you worked *when your youngest child was being breastfed*? আপনার সব থেকে ছোট বাচ্চাকে বুকের দুধ খাওয়ানোর সময় আপনি কতটা কাজ করেছেন অথবা করছেন? |  |  |  | Not at all একটুও নয়…1  Small extent অল্প পরিমাণ...2  Medium extent মাঝারি পরিমাণ...3  To a high extent উচ্চ মাত্রায় …4 | Not at all একটুও নয় …1  Somewhat কিছুটা 2  Very confident একেবারে অসংশয়/আত্মবিশ্বাসী 3 |  | |  |  |
| **L** | How much you could rest *when your youngest child was being breastfed*? আপনার সব থেকে ছোট বাচ্চাকে বুকের দুধ খাওয়ানোর সময় আপনি কতটা বিশ্রাম নিতে পেরেছিলেন অথবা পারছেন? |  |  |  | Not at all একটুও নয়…1  Small extent অল্প পরিমাণ...2  Medium extent মাঝারি পরিমাণ...3  To a high extent উচ্চ মাত্রায় …4 | Not at all একটুও নয় …1  Somewhat কিছুটা 2  Very confident একেবারে অসংশয়/আত্মবিশ্বাসী 3 |  | |  |  |
| **M** | Whether you could eat eggs *when your youngest child was being breastfed*? আপনার সব থেকে ছোট বাচ্চাকে বুকের দুধ খাওয়ানোর সময় আপনি কি ডিম খেতে পেরেছিলেন/পেয়েছিলেন অথবা পারছেন/পাচ্ছেন? |  |  |  | Not at all একটুও নয়…1  Small extent অল্প পরিমাণ...2  Medium extent মাঝারি পরিমাণ...3  To a high extent উচ্চ মাত্রায় …4 | Not at all একটুও নয় …1  Somewhat কিছুটা 2  Very confident একেবারে অসংশয়/আত্মবিশ্বাসী 3 |  | |  |  |
| **N** | Whether you could consume milk or milk products *when your youngest child was being breastfed*? আপনার সব থেকে ছোট বাচ্চাকে বুকের দুধ খাওয়ানোর সময় আপনি কি দুধ এবং দুধ জাত খাদ্য খেতে পেরেছিলেন/পেয়েছিলেন অথবা পারছেন/পাচ্ছেন? |  |  |  | Not at all একটুও নয়…1  Small extent অল্প পরিমাণ...2  Medium extent মাঝারি পরিমাণ...3  To a high extent উচ্চ মাত্রায় …4 | Not at all একটুও নয় …1  Somewhat কিছুটা 2  Very confident একেবারে অসংশয়/আত্মবিশ্বাসী 3 |  | |  |  |
| **O** | Whether you could eat meat, poultry or fish *when your youngest child was being breastfed*? আপনার সব থেকে ছোট বাচ্চাকে বুকের দুধ খাওয়ানোর সময় আপনি কি মাছ- মাংস (সব রকম)খেতে পেরেছিলেন/পেয়েছিলেন অথবা পারছেন/পাচ্ছেন? |  |  |  | Not at all একটুও নয়…1  Small extent অল্প পরিমাণ...2  Medium extent মাঝারি পরিমাণ...3  To a high extent উচ্চ মাত্রায় …4 | Not at all একটুও নয় …1  Somewhat কিছুটা 2  Very confident একেবারে অসংশয়/আত্মবিশ্বাসী 3 |  | |  |  |

**MODULE G11.1 NUTRITION AND HEALTH: ABOUT THE YOUNGEST CHILD** পুষ্টি এবং স্বাস্থ্য: সব থেকে ছোট বাচ্চার বিষয়ে

| The next set of questions asks about *making decisions about* ***your YOUNGEST child. পরবর্তী প্রশ্নগুলি আপনার*** সব থেকে ছোট বাচ্চার বিষয়ে আপনার সিধান্ত/নির্ণয় নেওয়া সংক্রান্ত  [Note for survey adaptation: Based on specific needs, projects may wish to provide more detailed response categories for **other non-household family members** (response code 6) listed for question GX.05 and GX.07. Example additions might include, RESPONDENT’S MOTHER (if outside household)…81; RESPONDENT’S MOTHER-IN-LAW (if outside household)…82; A SENIOR CO-WIFE (if outside household)…83; ABSENT HUSBAND…84. These response codes should be in the 80s.] | | Who is it that is normally involved in making decisions about [ACTIVITY]?  সাধারণত এই বিষয়ে নির্ণয় কে বা কাঁরা নিয়ে থাকেন?  **ENTER UP TO THREE (3) CODES তিনটি কোড অবধি ব্যাবহার করতে পারেন**  **IF RESPONSE IS MEMBER ID (SELF) ONLY** 🡪 **G11.03** উত্তর যদি নিজস্ব হয়, G11.03 তে যান  **CODES:**  Self নিজে …………1  Partner/Spouse in HH পরিবারে বসবাসকারী সঙ্গী/স্বামী/স্ত্রী …………2  Partner/Spouse outside HH পরিবারের থেকে দূরে থাকা সঙ্গী/স্বামী/স্ত্রী …………3  Other HH member পরিবারের অন্য সদস্য…………………4  NON-HH, NON-FAMILY MEMBER পরিবারের বাইরে থাকা ব্যক্তি যে পরিবারের সদস্য নন 5  Non-hh, FAMILY MEMBER পরিবারের বাইরে থাকা ব্যক্তি যে পরিবারের সদস্য 6  NOT APPLICABLE প্রযোজ্য নয়/ সিদ্ধান্ত নেওয়া হয়নি 98 >> NEXT Row পরের বিষয় | | | To what extent do you participate in decisions regarding [ACTIVITY]?  এই বিষয়ে **সিদ্ধান্ত** নেয়ায় আপনি কতটা অংশগ্রহণ করেন?  **CIRCLE ONE**  যেকোন একটিকে চিহ্নিত করুন | How confident do you feel to make decisions about [ACTIVITY]?  এই বিষয়ে **সিদ্ধান্ত** নিতে আপনি কতটা অসংশয়/আত্মবিশ্বাসী বোধ করেন?  **CIRCLE ONE**  যেকোন একটিকে চিহ্নিত করুন | When decisions are made regarding [ACTIVITY], who would you prefer made the decision?  আপনার মতে কার বা কাদের এই বিষয়ে **সিদ্ধান্ত নেওয়া উচিত**?  **ENTER UP TO THREE (3) CODES** তিনটি কোড অবধি ব্যাবহার করতে পারেন  **CODES:**  Self নিজে …………1  Partner/Spouse in HH পরিবারে বসবাসকারী সঙ্গী/স্বামী/স্ত্রী …………2  Partner/Spouse outside HH পরিবারের থেকে দূরে থাকা সঙ্গী/স্বামী/স্ত্রী …………3  Other HH member পরিবারের অন্য সদস্য…………………4  NON-HH, NON-FAMILY MEMBER পরিবারের বাইরে থাকা ব্যক্তি যে পরিবারের সদস্য নন 5  Non-hh, FAMILY MEMBER পরিবারের বাইরে থাকা ব্যক্তি যে পরিবারের সদস্য 6  NOT APPLICABLE প্রযোজ্য নয়/ সিদ্ধান্ত নেওয়া হয়নি 98 >> NEXT Row পরের বিষয় | | | | |
| --- | --- | --- | --- | --- | --- | --- | --- | --- | --- | --- | --- |
| **CHILD HEALTH AND NUTRITION** | | **G11.06** | | | **G11.07** | **G11.08** | **G11.09** | | | | |
|  |  | **ID #1** | **ID #2** | **ID #3** |  |  | **ID #1** | **ID #2** | | **ID #3** | |
| **A** | Whether your youngest child is taken to a clinic or a doctor is consulted when he/she is sick? আপনার সব থেকে ছোট বাচ্চার শরীর খারাপ হলে তাকে আপনি ডাক্তারের কাছে অথবা কোন স্বাস্থ্য কেন্দ্রে নিয়ে যাওয়া হবে কিনা? |  |  |  | Not at all একটুও নয়…1  Small extent অল্প পরিমাণ...2  Medium extent মাঝারি পরিমাণ...3  To a high extent উচ্চ মাত্রায় …4 | Not at all একটুও নয় …1  Somewhat কিছুটা 2  Very confident একেবারে অসংশয়/আত্মবিশ্বাসী 3 |  | |  | |  |
| **B** | Whether your youngest child gets vaccinations? আপনার সব থেকে ছোট বাচ্চাকে টিকা দেয়া হবে কিনা? |  |  |  | Not at all একটুও নয়…1  Small extent অল্প পরিমাণ...2  Medium extent মাঝারি পরিমাণ...3  To a high extent উচ্চ মাত্রায় …4 | Not at all একটুও নয় …1  Somewhat কিছুটা 2  Very confident একেবারে অসংশয়/আত্মবিশ্বাসী 3 |  | |  | |  |
| **C** | Whether your youngest child visits the health clinic to see if he/she is growing well? আপনার সব থেকে ছোট বাচ্চা সঠিক ভাবে বাড়ছে কিনা তা যাচাই এর জন্য স্বাস্থ্য কেন্দ্রে নিয়ে যাওয়া হবে কিনা? |  |  |  | Not at all একটুও নয়…1  Small extent অল্প পরিমাণ...2  Medium extent মাঝারি পরিমাণ...3  To a high extent উচ্চ মাত্রায় …4 | Not at all একটুও নয় …1  Somewhat কিছুটা 2  Very confident একেবারে অসংশয়/আত্মবিশ্বাসী 3 |  | |  | |  |
| **D** | Who will care for your youngest child when you need to go outside the home for an extended period of time? আপনাকে যদি বাড়ির বাইরে দীর্ঘ সময়ের জন্য যেতে হয়, আপনার সব থেকে ছোট বাচ্চার দেখাশোনা কে করবেন? |  |  |  | Not at all একটুও নয়…1  Small extent অল্প পরিমাণ...2  Medium extent মাঝারি পরিমাণ...3  To a high extent উচ্চ মাত্রায় …4 | Not at all একটুও নয় …1  Somewhat কিছুটা 2  Very confident একেবারে অসংশয়/আত্মবিশ্বাসী 3 |  | |  | |  |
| **E** | (If youngest child is >6 months of age:) (যদি আপনার সব থেকে ছোট বাচ্চার বয়স 6 মাস অথবা তার বেশি হয়ে থাকে)  Whether or not your youngest child is offered eggs to eat? আপনার সব থেকে ছোট বাচ্চার ডিম খেতে দেওয়া হবে কিনা? |  |  |  | Not at all একটুও নয়…1  Small extent অল্প পরিমাণ...2  Medium extent মাঝারি পরিমাণ...3  To a high extent উচ্চ মাত্রায় …4 | Not at all একটুও নয় …1  Somewhat কিছুটা 2  Very confident একেবারে অসংশয়/আত্মবিশ্বাসী 3 |  | |  | |  |
| **F** | (If youngest child is >6 months of age:) (যদি আপনার ক সব থেকে ছোট বাচ্চার বয়স 6 মাস অথবা তার বেশি হয়ে থাকে)  Whether or not your youngest child is offered milk or milk products, other than breastmilk? আপনার কনিষ্ঠ সন্তানকে মায়ের দুধ ছাড়া দুধ এবং দুধ জাত খাদ্য খেতে দেওয়া হবে কিনা? |  |  |  | Not at all একটুও নয়…1  Small extent অল্প পরিমাণ...2  Medium extent মাঝারি পরিমাণ...3  To a high extent উচ্চ মাত্রায় …4 | Not at all একটুও নয় …1  Somewhat কিছুটা 2  Very confident একেবারে অসংশয়/আত্মবিশ্বাসী 3 |  | |  | |  |
| **G** | (If youngest child is >6 months of age:) (যদি আপনার সব থেকে ছোট বাচ্চার বয়স 6 মাস অথবা তার বেশি হয়ে থাকে)  Whether or not your youngest child if offered meat, poultry or fish? আপনার সব থেকে ছোট বাচ্চার মাছ-মাংস খেতে দেওয়া হবে কিনা? |  |  |  | Not at all একটুও নয়…1  Small extent অল্প পরিমাণ...2  Medium extent মাঝারি পরিমাণ...3  To a high extent উচ্চ মাত্রায় …4 | Not at all একটুও নয় …1  Somewhat কিছুটা 2  Very confident একেবারে অসংশয়/আত্মবিশ্বাসী 3 |  | |  | |  |
| **G11.10** Do you have a child less than 4 years of age*? আপনার কি 4 বছর বয়েসের কমে কোন সন্তান আছে?  [Note for survey adaptation: The timeframe highlighted for **G11.10** should be altered to reflect the program implementation period, such that women are only being asked this question if the child was born after the start of program implementation. Ideally, this same timeframe should be used at all surveys.] | | | | | | | YES হ্যাঁ...1  NO না… 2 🡪 ***G11.11*** | | | | |
| **H** | Whether to breastfeed your child? আপনার বাচ্চাকে বুকের দুধ খাওয়ানো হবে কিনা? |  |  |  | Not at all একটুও নয়…1  Small extent অল্প পরিমাণ...2  Medium extent মাঝারি পরিমাণ...3  To a high extent উচ্চ মাত্রায় …4 | Not at all একটুও নয় …1  Somewhat কিছুটা 2  Very confident একেবারে অসংশয়/আত্মবিশ্বাসী 3 |  | |  | |  |
| **I** | When to stop breastfeeding your child? আপনার বাচ্চাকে বুকের দুধ খাওয়ানো কখন বন্ধ করা হবে? |  |  |  | Not at all একটুও নয়…1  Small extent অল্প পরিমাণ...2  Medium extent মাঝারি পরিমাণ...3  To a high extent উচ্চ মাত্রায় …4 | Not at all একটুও নয় …1  Somewhat কিছুটা 2  Very confident একেবারে অসংশয়/আত্মবিশ্বাসী 3 |  | |  | |  |
| **J** | When to start giving foods and liquids (other than breastmilk) to your child? আপনার বাচ্চাকে (বুকের দুধ ছাড়া) তরল এবং শক্ত খাদ্য খাওয়ানো কবে থেকে শুরু করা হবে? |  |  |  | Not at all একটুও নয়…1  Small extent অল্প পরিমাণ...2  Medium extent মাঝারি পরিমাণ...3  To a high extent উচ্চ মাত্রায় …4 | Not at all একটুও নয় …1  Somewhat কিছুটা 2  Very confident একেবারে অসংশয়/আত্মবিশ্বাসী 3 |  | |  | |  |

**MODULE G11.2 NUTRITION AND HEALTH: OBTAIN FOOD, MEDICINE AND OTHER ITEMS** পুষ্টি এবং স্বাস্থ্য: খাদ্য, ঔষধ এবং অন্য জিনিসপত্র পাওয়ার বিষয়ে

| The next set of questions asks about making decisions and your ability to obtain the types of food, medicine and other items that you want for you and your child/children. এই প্রশ্নগুলো আপনার এবং আপনার বাচ্চাদের বিভিন্ন প্রকারের খাদ্য, ঔষধ এবং অন্য জিনিসপত্র পাওয়া এবং সেই বিষয়ে সিধান্ত/নির্ণয় নেওয়ার সম্পর্কে।  [Note for survey adaptation: Based on specific needs, projects may wish to provide more detailed response categories for **other non-household family members** (response code 6) listed for question GX.09. Example additions might include, RESPONDENT’S MOTHER (if outside household)…81; RESPONDENT’S MOTHER-IN-LAW (if outside household)…82; A SENIOR CO-WIFE (if outside household)…83; ABSENT HUSBAND…84. These response codes should be in the 80s.] | | Who is normally involved about whether or not to purchase these [PRODUCT]? কে অথবা কারা সাধারনত [জিনিস] কেনা বা না কেনার সিধান্ত/নির্ণয় নিয়ে থাকেন?  **ENTER UP TO THREE (3) CODES তিনটি কোড অবধি ব্যাবহার করতে পারেন**  **OTHER CODES:**  Self নিজে …………1  Partner/Spouse in HH পরিবারে বসবাসকারী সঙ্গী/স্বামী/স্ত্রী …………2  Partner/Spouse outside HH পরিবারের থেকে দূরে থাকা সঙ্গী/স্বামী/স্ত্রী …………3  Other HH member পরিবারের অন্য সদস্য…………………4  NON-HH, NON-FAMILY MEMBER পরিবারের বাইরে থাকা ব্যক্তি যে পরিবারের সদস্য নন 5  Non-hh, FAMILY MEMBER পরিবারের বাইরে থাকা ব্যক্তি যে পরিবারের সদস্য 6  NOT APPLICABLE প্রযোজ্য নয়/ সিদ্ধান্ত নেওয়া হয়নি 98 >> NEXT Row পরের জিনিস | | | You may acquire an item that you need in a variety of ways, such as purchasing or cultivating it or having someone purchase or cultivate it for you. When you need these [PRODUCT], can you usually acquire it? আপনি বিভিন্ন উপায়ে যে কোনো প্রয়োজনীয় জিনিস পেতে পারেন যেমন কিনে, চাষ করে অথবা কেউ আপনাকে কিনে দিলেন বা চাষ করে দিলেন। আপনি কি সাধারনত প্রয়োজনীয় [জিনিস] পান? |
| --- | --- | --- | --- | --- | --- |
|  | | **G11.11** | | | **G11.12** |
|  |  | **ID #1** | **ID #2** | **ID #3** |  |
| **A** | Small amounts of food, for example smaller than 5 kg অল্প পরিমান খাবার যা 5 কেজির থেকে কম |  |  |  | Yes হ্যাঁ 1  NO না 2  NOT APPLICABLE প্রযোজ্য নয় 98 |
| **B** | Larger amounts of food, for example larger than 5 kg বেশি পরিমান খাবার যা 5 কেজির থেকে বেশী |  |  |  | Yes হ্যাঁ 1  NO না 2  NOT APPLICABLE প্রযোজ্য নয় 98 |
| **C** | Eggs ডিম |  |  |  | Yes হ্যাঁ 1  NO না 2  NOT APPLICABLE প্রযোজ্য নয় 98 |
| **D** | Milk or milk products দুধ এবং দুধ থেকে তৈরি জিনিস |  |  |  | Yes হ্যাঁ 1  NO না 2  NOT APPLICABLE প্রযোজ্য নয় 98 |
| **E** | Meat, poultry or fish (including organ meats) মাংস, মুরগির মাংস, মাছ এবং মেটে বা ওই ধরনের মাংস যা একটা পশুপাখির বিশেষ অঙ্গ থেকে পাওয়া যায় |  |  |  | Yes হ্যাঁ 1  NO না 2  NOT APPLICABLE প্রযোজ্য নয় 98 |
| **F** | Any nutritious foods that a program or health worker told you to eat or drink যে কোন পুষ্টিকর খাবার যা কোন স্বাস্থ্য কর্মী অথবা কোন প্রোগ্রামের মাধ্যমে আপনাকে খেতে বলা হয়েছে। |  |  |  | Yes হ্যাঁ 1  NO না 2  NOT APPLICABLE প্রযোজ্য নয় 98 |
| **G** | Medication, vitamins or supplements for children বাচ্চাদের ঔষধ, ভিটামিন এবং সাধারণ খাবারের বাইরে পুষ্টি বাড়ানোর জন্য বিশেষ ভাবে তৈরি খাবার |  |  |  | Yes হ্যাঁ 1  NO না 2  NOT APPLICABLE প্রযোজ্য নয় 98 |
| **H** | Medication, vitamins or supplements for yourself নিজের জন্য ঔষধ, ভিটামিন এবং সাধারণ খাবারের বাইরে পুষ্টি বাড়ানোর জন্য বিশেষ ভাবে তৈরি খাবার |  |  |  | Yes হ্যাঁ 1  NO না 2  NOT APPLICABLE প্রযোজ্য নয় 98 |
| **I** | Toiletries, such as soap and toothpaste প্রসাধন সামগ্রী যেমন সাবান, টুথ-পেস্ট (মাঞ্জন) |  |  |  | Yes হ্যাঁ 1  NO না 2  NOT APPLICABLE প্রযোজ্য নয় ......98 |

**MODULE G12: Time allocation *সময় বরাদ্দ***

**G12.01: PLEASE RECORD A LOG OF THE ACTIVITIES FOR THE INDIVIDUAL IN THE LAST COMPLETE 24 HOURS (STARTING YESTERDAY MORNING AT 4 AM, FINISHING 3:59 AM OF THE CURRENT DAY). THE TIME INTERVALS ARE MARKED IN 15 MIN INTERVALS. MARK ONE ACTIVITY FOR EACH TIME PERIOD BY ENTERING THE CORRESPONDING ACTIVITY CODE IN THE BOX.**

দয়া করে সর্বশেষ 24 ঘন্টার মধ্যে প্রতিটি স্বতন্ত্রের জন্য ক্রিয়াকলাপগুলির একটি লগ রেকর্ড করুন, শুরু হচ্ছে গতকাল ভোর চারটায়, সমাপ্তি বর্তমান দিনের ০৩:৫৯ টায়। সময় অন্তর ১৫ মিনিটের ব্যবধানে চিহ্নিত করা হয়েছে। বাক্সে সংশ্লিষ্ট ক্রিয়াকলাপ কোড প্রবেশ করিয়ে প্রতিটি সময়কালের জন্য একটি ক্রিয়াকলাপ চিহ্নিত করুন।

**G12.02: CHECK THE BOX BELOW IF THE RESPONDENT WAS CARING FOR CHILDREN WHILE PERFORMING EACH ACTIVITY.**

উত্তরদাতা প্রতিটি ক্রিয়াকলাপ সম্পাদন করার সময় বাচ্চাদের যত্ন নিচ্ছে কিনা তা নীচের বাক্সটি চেক করুন।

Now I ‘d like to ask you how you spent your time during the past 24 hours. We will begin from yesterday morning and continue through this morning. This will be a detailed accounting. I am interested in everything you did (i.e. resting, eating, personal care, work inside and outside the house, caring for children, cooking, shopping, socializing, etc.) even if it did not take you much time. I am particular interested in agricultural and fishing activities. I am also interested in how much time you spent caring for children, especially if it happened while you did some other activity.

**এখন আমি আপনাকে জিজ্ঞাসা করতে চাই যে আপনি কীভাবে গত ২৪ ঘন্টা সময় কাটিয়েছেন? আমরা গতকাল সকাল থেকে শুরু করব এবং এই সকাল দিয়ে চালিয়ে যাব। এটি একটি বিস্তারিত অ্যাকাউন্টিং (হিসাব) হবে। আপনার করা সমস্ত কিছুতে আমি আগ্রহী (যেমন বিশ্রাম নেওয়া, খাওয়া, ব্যক্তিগত যত্ন, বাড়ির অভ্যন্তরে এবং বাইরে কাজ করা, বাচ্চাদের যত্ন নেওয়া, রান্না করা, কেনাকাটা করা, সামাজিককরণ ইত্যাদি), এই কাজগুলিতে আপনি বেশি সময় না নিলেও। আমি কৃষি এবং মাছধরা কার্যক্রমগুলিতে বিশেষ আগ্রহী। আপনি বাচ্চাদের যত্ন নেওয়ার জন্য কতটা সময় ব্যয় করেছেন সে সম্পর্কেও আমি আগ্রহী, বিশেষত যদি আপনি অন্য কোনও ক্রিয়াকলাপ করার সময় এটি করে থাকেন।**

| **Code 6** | | |
| --- | --- | --- |
| **Personal and leisure activities**  **ব্যক্তিগত কাজ এবং অবসর সময়** | **Domestic work activities** গার্হস্থ্য (বাড়ির) কাজ-কর্ম | **Work and school activities** উপার্জনের (রোজগার) এবং পড়াশোনার সঙ্গে যুক্ত কাজ-কর্ম |
| **Sleeping and resting ঘুম ও বিশ্রাম…………..P1**  **Eating and drinking খাওয়া-দাওয়া …….....P2**  **Personal care ব্যক্তিগত শারীরিক যত্ন ………..…P3**  **Exercising ব্যয়াম শরীর চর্চা …..……P4**  **Religious activities ধর্মীয় ক্রিয়াকলাপ………….…P5**  **Traveling (not for work or school) ভ্রমণ (কাজের বা পড়াশোনার ছাড়া) …………………….…….….P6**  **Social activities and hobbies (incl. entertainment, watching tv, social media / gaming, reading) সামাজিক মেলামেশা এবং শখ (বিনোদন, টিভি দেখা, সামাজিক মিডিয়া/গেম খেলা, পড়া সহ)………….P7** | **Cooking and food preparation রান্না এবং খাবার প্রস্তুতি.….D1**  **Caring for childrenবাচ্চাদের যত্ন নেওয়া………………..….D2**  **Caring for adults (sick, elderly) প্রাপ্তবয়স্কদের (অসুস্থ, বয়স্ক) যত্ন নেওয়া ……………………………………..D3**  **Domestic work (household chores incl. fetching water and collecting fuel) গৃহস্থালি কাজ (গৃহস্থালী কাজগুলি আনয়ন সহ**  **জল এবং জ্বালানী সংগ্রহ) ………….....D4**  **Shopping / getting service (including health services) কেনাকাটা / সেবা প্রাপ্তি (স্বাস্থ্যসেবা সহ) ………...…D5**  **Weaving / sewing for home useঘরের ব্যবহারের জন্য বুনন / সেলাই ………………………………….....D6**  **Hunting / gathering for home use d ঘরের ব্যবহারের জন্য শিকার / জমায়েত ………………………………….D7** | **Work as employed (farm & non-farm wage or salary employment) মাইনে বা মজুরি ভিত্তিক কাজ (কৃষি বা অন্য ক্ষেত্রে মজুরি বা বেতন ভিত্তিক কর্মসংস্থান)………………W1**  **Work in own business (own or household non-farm business work) নিজস্ব ব্যবসার কাজ (কৃষি-ব্যবসা ছাড়া ………….W2**  **Staple grain farming (general) প্রধান শস্য চাষ (সাধারণ)…...........W3**  **Horticultural (gardens) or high value crop farming উদ্যান (বাগিচা) বা উচ্চমূল্যের ফসলের চাষ ……..….W4**  **Large livestock raising (cattle, buffaloes) বড় পশুপালন (গবাদি পশু, মহিষ)……...….W5**  **Small livestock raising (sheep, goats, pigs) ছোট পশু পালন (ভেড়া, ছাগল, শূকর) ………...W6**  **Poultry and other small animals raising (chickens, geese, ducks, turkeys, quail, rabbits) হাঁস-মুরগি এবং অন্যান্য ছোট প্রাণী পালন (মুরগী, রাজ হাঁস, হাঁস, টার্কি, কোয়েল, খরগোশ)…...W7**  **Fishpond culture পুকুরে মাছ চাষ …..….W8**  **Commuting (to/from work or school) কাজের জায়গা বা স্কুলে যাতায়াত ..….W9**  **School / training (including homework) স্কুল / প্রশিক্ষণ (হোম-ওয়ার্ক বা বাড়ির পড়া সহ)………….………………….W10**  **Other activities** অন্যান্য কাজ-কর্ম  **Other (specify) অন্যান্য কাজ (বিবরণ)………….….O1**  **Other (specify) অন্যান্য কাজ (বিবরণ) ……..…….O2**  **Other (specify) অন্যান্য কাজ (বিবরণ)………….…. O3** |

| **MODULE G12: Time allocation** | **G12.03** |
| --- | --- |
|  | In order to help me understand if this day (above) is usual for you or not, in the last 24 hours, did you work (at home or outside of the home including chores or other domestic activities) less than usual, more than usual, or about the same?  গত ২৪ ঘন্টায় আপনি কি (বাড়ির বা বাড়ির বাইরের কাজ) স্বাভাবিকের চেয়ে কম না বেশী না একই প্রকার কাজ করেছেন ? এটা আমাকে বুঝতে সাহায্য করবে যে এই দিনটি আপনার জন্য স্বাভাবিক বা স্বাভাবিক নয়। |
|  | Less than usual স্বাভাবিকের চেয়ে কম……1  More than usual স্বাভাবিকের চেয়ে বেশি……..2  About the same প্রায় একই…………………..3 |
|  |  |
