# Supplementary material for: Measuring women’s empowerment in aquaculture in northwestern Bangladesh using a project level women’s empowerment in fisheries index (pro-WEFI)
Source: PLoS One. 2025 Jan 10;20(1):e0314284. doi: 10.1371/journal.pone.0314284 (PMC11723601; doi:10.1371/journal.pone.0314284)
Supplement: Attachment — Submitted filename: PONE-D-23-09595_reviewer comments.pdf [file pone.0314284.s003.pdf]

PLOS ONE

The state of women’s empowerment in the aquaculture sector: the case of Bangladesh

--Manuscript Draft--

**Manuscript Number:**	PONE-D-23-09595
**Article Type:**	Research Article
**Full Title:**	The state of women’s empowerment in the aquaculture sector: the case of Bangladesh
**Short Title:**	Women’s empowerment in fisheries and aquaculture index
**Corresponding Author:**	Rahma Adam<br>WorldFish Center: WorldFish<br>Nairobi, KENYA
**Keywords:**	Women's empowerment, gender disparities, aquaculture, Bangladesh and agency.
**Abstract:**	To be able to determine the impact of projects that focuses on fisheries and/or aquaculture on women’s empowerment, agency, it is crucial to have a dependable instrument to collect data with. As a result of this need, a tool has been developed, called the project level women’s empowerment in fisheries and aquaculture index (pro-WEFI). The instrument bears its roots from the project-level women’s empowerment in agriculture index (pro-WEAI). We implemented the pro-WEFI in 217 households that are engaged in aquaculture in northwestern Bangladesh, in order to determine how gender equity in, for instance, decision making in productive activities, asset ownership, gender attitudes, among others is different and/or similar for women and men. We found that, for the sampled women, inability to visit important locations, lack of ownership of land and other assets, lack of access to credit, not being a member to influential groups are most disempowering aspects for women in those respective households.
**Order of Authors:**	Rahma Adam
	Surendran Rajaratnam, PhD
	Farha Deba Sufian, Masters
	Lucy Njogu
**Additional Information:**	
**Question**	**Response**
**Financial Disclosure**<br><br>Enter a financial disclosure statement that describes the sources of funding for the work included in this submission. Review the submission guidelines for detailed requirements. View published research articles from *PLOS ONE* for specific examples.<br><br>This statement is required for submission and **will appear in the published article** if the submission is accepted. Please make sure it is accurate.	Bill and Melinda Gates Foundation<br>CGIAR Gender Impact platform<br>The funders had no role in study design, data collection and analysis, decision to publish, or preparation of the manuscript.

Powered by Editorial Manager® and ProduXion Manager® from Aries Systems Corporation

**Unfunded studies**<br>Enter: *The author(s) received no specific funding for this work.*<br><br>**Funded studies**<br>Enter a statement with the following details:<br>• Initials of the authors who received each award<br>• Grant numbers awarded to each author<br>• The full name of each funder<br>• URL of each funder website<br>• Did the sponsors or funders play any role in the study design, data collection and analysis, decision to publish, or preparation of the manuscript?<br>• **NO** - Include this sentence at the end of your statement: *The funders had no role in study design, data collection and analysis, decision to publish, or preparation of the manuscript.*<br>• **YES** - Specify the role(s) played.<br><br>* typeset	
**Competing Interests**<br><br>Use the instructions below to enter a competing interest statement for this submission. On behalf of all authors, disclose any competing interests that could be perceived to bias this work—acknowledging all financial support and any other relevant financial or non-financial competing interests.<br><br>This statement is required for submission and **will appear in the published article** if the submission is accepted. Please make sure it is accurate and that any funding sources listed in your Funding Information later in the submission form are also declared in your Financial Disclosure statement.<br><br>View published research articles from *PLOS ONE* for specific examples.	The authors have declared that no competing interests exist.

**NO authors have competing interests**<br><br>Enter: *The authors have declared that no competing interests exist.*<br><br>**Authors with competing interests**<br><br>Enter competing interest details beginning with this statement:<br><br>*I have read the journal's policy and the authors of this manuscript have the following competing interests: [insert competing interests here]*<br><br>* typeset	
**Ethics Statement**<br><br>Enter an ethics statement for this submission. This statement is required if the study involved:<br><br>• Human participants<br>• Human specimens or tissue<br>• Vertebrate animals or cephalopods<br>• Vertebrate embryos or tissues<br>• Field research<br><br>Write "N/A" if the submission does not require an ethics statement.<br><br>General guidance is provided below. Consult the submission guidelines for detailed instructions. **Make sure that all information entered here is included in the Methods section of the manuscript.**	Institute of Health Economics, University of Dhaka authorized this study to take place in Bangladesh. The Ref No IHE/IRB/DU/22/2022/Final.<br>The form of consent was oral.

Powered by Editorial Manager® and ProduXion Manager® from Aries Systems Corporation

**Format for specific study types**<br><br>**Human Subject Research (involving human participants and/or tissue)**<br>• Give the name of the institutional review board or ethics committee that approved the study<br>• Include the approval number and/or a statement indicating approval of this research<br>• Indicate the form of consent obtained (written/oral) or the reason that consent was not obtained (e.g. the data were analyzed anonymously)<br><br>**Animal Research (involving vertebrate animals, embryos or tissues)**<br>• Provide the name of the Institutional Animal Care and Use Committee (IACUC) or other relevant ethics board that reviewed the study protocol, and indicate whether they approved this research or granted a formal waiver of ethical approval<br>• Include an approval number if one was obtained<br>• If the study involved *non-human primates*, add *additional details* about animal welfare and steps taken to ameliorate suffering<br>• If anesthesia, euthanasia, or any kind of animal sacrifice is part of the study, include briefly which substances and/or methods were applied<br><br>**Field Research**<br><br>Include the following details if this study involves the collection of plant, animal, or other materials from a natural setting:<br>• Field permit number<br>• Name of the institution or relevant body that granted permission	
**Data Availability**<br><br>Authors are required to make all data underlying the findings described fully available, without restriction, and from the time of publication. PLOS allows rare exceptions to address legal and ethical concerns. See the PLOS Data Policy and FAQ for detailed information.	No - some restrictions will apply

A Data Availability Statement describing where the data can be found is required at submission. Your answers to this question constitute the Data Availability Statement and **will be published in the article**, if accepted.<br><br>**Important:** Stating 'data available on request from the author' is not sufficient. If your data are only available upon request, select 'No' for the first question and explain your exceptional situation in the text box.<br><br>Do the authors confirm that all data underlying the findings described in their manuscript are fully available without restriction?	
**Describe where the data may be found in full sentences. If you are copying our sample text, replace any instances of XXX with the appropriate details.**<br><br>• If the data are **held or will be held in a public repository**, include URLs, accession numbers or DOIs. If this information will only be available after acceptance, indicate this by ticking the box below. For example: *All XXX files are available from the XXX database (accession number(s) XXX, XXX.).*<br>• If the data are all contained **within the manuscript and/or Supporting Information files**, enter the following: *All relevant data are within the manuscript and its Supporting Information files.*<br>• If neither of these applies but you are able to provide **details of access elsewhere**, with or without limitations, please do so. For example:<br><br>*Data cannot be shared publicly because of [XXX]. Data are available from the XXX Institutional Data Access / Ethics Committee (contact via XXX) for researchers who meet the criteria for access to confidential data.*<br><br>*The data underlying the results presented in the study are available from (include the name of the third party*	Data is available on WorldFish MELIA repository, but it is restricted to WorldFish staff, until we have published the papers, and at least one year and a half have passed since we have collected the data. But, if needed to check the validity of our findings or for integrity purpose, they can be shared without hesitation. Will need to reach out to Saadiah Ghazali at S.Ghazali@ cgiar.org

and contact information or URL).<br>• This text is appropriate if the data are owned by a third party and authors do not have permission to share the data.<br><br>* typeset	
Additional data availability information:	Tick here if the URLs/accession numbers/DOIs will be available only after acceptance of the manuscript for publication so that we can ensure their inclusion before publication.

Manuscript

Click here to access/download;Manuscript;Final Blind Pro-WEFI Bangladesh validation_30.3.2023.docx

The state of women’s empowerment in the aquaculture sector: the case of Bangladesh

Abstract

To be able to determine the impact of projects that focuses on fisheries and/or aquaculture on women’s empowerment, agency, it is crucial to have a dependable instrument to collect data with. As a result of this need, a tool has been developed, called the project level women’s empowerment in fisheries and aquaculture index (pro-WEFI). The instrument bears its roots from the project-level women’s empowerment in agriculture index (pro-WEAI). We implemented the pro-WEFI in 217 households that are engaged in aquaculture in northwestern Bangladesh, in order to determine how gender equity in, for instance, decision making in productive activities, asset ownership, gender attitudes, among others is different and/or similar for women and men. We found that, for the sampled women, inability to visit important locations, lack of ownership of land and other assets, lack of access to credit, not being a member to influential groups are most disempowering aspects for women in those respective households.

Key words: Women’s empowerment, gender disparities, aquaculture, Bangladesh and agency.

1. Introduction

The Sustainable Development Goal number five, which is to achieve gender equality and empower all women and girls has an intrinsic value to fundamental human rights. Fulfilment of women’s human rights supports women in developing a sense of self-worth and confidence to explore their potential (Bayeh, 2016) and (Diiro et al., 2018). Women’s empowerment and gender equality is linked to attainment of other sustainable development goals (Rettig et al., 2020). For example, research shows that increase in women’s empowerment has a positive

effect on children's nutritional security (Heckert et al., 2019; Malapit et al., 2015). Similarly, Bayeh, (2016) demonstrates that without women's empowerment a country's goals towards economic growth, social development, human right and environmental protection, are repressed.

The pivotal role of women's empowerment in the agricultural economic activity, cannot be over-emphasized, given that the sector plays a pivotal role in most developing countries' economies. Improvement of women's empowerment leads to positive changes in agricultural production (Anik & Rahman, 2021; Diiro et al., 2018; Mobarok et al., 2021). In fisheries and aquaculture, women provide nearly half of the labor employed worldwide. Income derived from fisheries and aquaculture by women significantly contributes to household's income and food security (UN Women, 2020). On the other hand, literature indicates that women's engagement in fisheries and aquaculture leads to their empowerment (Rahman & Naoroze, 2007; Torre et al., 2019). However, women's work in this subsector under-recognized, resulting to their exclusion from making decisions (Choudhury et al., 2017; Torre et al., 2019; UN Women, 2020). Lack of proper data collection and analysis of women in fisheries and aquaculture, partly due to lack of comprehensive data collection, analysis and gender focused evaluation methodologies, significantly drives their exclusion from development projects (Gonzalez Parrao et al., 2021; UN Women, 2020). So the same can be said to improving women's responsible and beneficial engagement in aquaculture and fisheries sector.

2. Background and motivation for creating and implementing pro-WEFI

Given the increasingly importance of the need to empower women and to reduce gender inequalities in accessing financial, physical (natural resources), assets, training, participation in food markets, calculating the level of empowerment of women in aquaculture and fisheries is crucial. It is important in informing national goals as well as development projects aimed at supporting women, children and men to move up the economic ladder. Following the increased

interest in reducing gender equality and women's empowerment, various diagnostic tools been developed. Women's empowerment literature has varying definitions of women's empowerment which leads to varying approaches to measurements (Alkire et al., 2013). However, there is general agreement that economic and social factors are key to women's empowerment (Bayissa et al., 2018), though the extent to which each measure incorporate these factors vary across different contexts. Kabeer, (1999), defines women's empowerment as a series of actions or steps that are taken in order to enlarge people's ability and capability to take key life decisions, specifically in areas where their ability has been rejected. This definition encompasses agency, resources, and achievements, but also includes agency (Quisumbing et al., 2021).

There are several composite indices that measure women's empowerment levels, while at the same time assessing the disparity between men and women; they include gender-based development index (GDI) and gender-based empowerment measure (GEM), gender gap index (GGI) and the gender inequality index (GII) (Mganga et al., 2021). To compute the GDI data on life expectancy, data on income, literacy and educational enrolment rates, is used while in development of GEM, data on employment status in higher positions, income and participation in political matters, is used (Klasen, 2006; Rettig et al., 2020). As for the GGI, data on economic participation and opportunity, educational attainment, health, and political participation is used (Worsdale & Wright, 2021). As noted by Alkire et al. (2013), the data used in GDI, GEM, GGI and GII are important in charting the way forward towards women's empowerment but the indicators are often proxying and not true measures of empowerment as experienced by individuals. Further, these indices, particularly the GDI and GEM are not easily interpretable and not internationally comparable (Klasen, 2006), use of major publicly available databases, and measure gender equality and women's empowerment at the aggregate country level. As a result, heterogeneities across regions, ethnicities, socioeconomic status,

marital status and age, are overlooked (Alkire et al., 2013a). Furthermore, majority of the gender equality indicators have minimal or no coverage or reference of the agricultural sector, making them un-ideal for analysis of women's empowerment in this sector and it's subsectors (Alkire et al., 2013a, Mganga et al., 2021).

The Women's Empowerment in Agriculture Index (WEAI) sharply contrasts with previous empowerment indices, addressing the previously highlighted shortcomings. The WEAI – which originates from Kabeer's definition of women's empowerment – includes social and economic domains which are similar to both woman and man in the household. It is rooted on a multi-dimensional poverty index (Alkire & Foster, 2011), but uses household survey data (Yount et al., 2019). It captures the gender parity and clearly indicates how various domains contribute to the (dis) empowerment of women and men in various economic categories. Agricultural activities are encompassed in all the domains, including productive and economic activities, as well as in decisions making. The WEAI can be used to measure empowerment for farming and non-farming respondents (Alkire et al., 2013b). To suit project needs and resource constraints a project Women's Empowerment Index in Agriculture (pro-WEAI) and Abbreviated Women's Empowerment in Agriculture Index (A-WEFI), have been developed and are available for use. The pro-WEAI is ideal for assessment of project impacts while A-WEAI is best suitable in time and funds constrained projects, as it is shorter and takes less time to administer (Yount et al., 2019).

Construction of the WEAI, as well as the pro-WEAI and A-WEAI, brought forth other sector specific derivatives. For instance, the Women Empowerment in Livestock Index (WELI) was derived for the livestock sector (Teufel et al., 2019) while Women's Empowerment in Nutrition Index (WENI) (Narayanan et al., 2019; Saha & Narayanan, 2022), was generated to address nutritional empowerment assessments and evaluations. Similarly, the Women's Empowerment in Fisheries and Aquaculture index (WEFI) and the project WEFI index (pro-WEFI), were

adapted from the WEAI and pro-WEAI, respectively, to small-scale fisheries and aquaculture context.

Specifically, the pro-WEFI was developed by adapting pro-WEAI and adjusting it to fit the contexts of aquaculture and small-scale fisheries. This was achieved through means of both qualitative and quantitative assessments of households who engaged in fishing and aquaculture. The WEAI has been in use since 2012 and it one of the most applied tool used to measure women's empowerment in the agriculture sector, it has a strong focus on crops (Colverson et al., 2020). To learn more about the five domains of empowerment in the WEAI (see Alkire et al., 2013), applied to measure intrinsic, instrumental, and collective agency. The pro-WEAI focuses more on the achievement goals set up by an agriculture development project. To learn more about it see (Yount et al., 2016; Cheong, Yount, and Crandall 2017; Miedema et al., 2018). To study women's empowerment in settings where fisheries and aquaculture is the primary economic means of livelihood, the project level Women's Empowerment in Fisheries index (pro-WEFI) was developed by WorldFish, in partnership with KIT Royal Tropical Institute and index experts from IFPRI. Like the WEAI and pro-WEAI, pro-WEFI is grounded on Kabeer's (1999, 2005) framework of empowerment. Empowerment is described as a process of change on the connected measures of agency, resources, and achievements. Moreover, it emphasized on measuring agency, or the ability of individuals, who engage in engage in fisheries and aquaculture, to make strategic choices. Creating a measure to depict the empowerment of women engaged in fisheries and aquaculture is vital to look at: 1) how fisheries and aquaculture and women's empowerment are related, especially how women's empowerment can be encouraged via agricultural activity, 2) what strategies worked to empower women in aquaculture and 3) unintended project impacts. Furthermore, the pro-WEFI differs from the WEAI, in that it adds fisheries or aquaculture related indicators in the survey. Specifically, for module 2, in the power/input in household decision making around production

and income generation in the fishing/aquaculture theme, the survey captures, fish farming homestead pond, fish farming commercial pond, in & off shore fishing; in the post fishing activities, the survey captures, fish processing/drying, fish storage, fish marketing and retailing. In module 3(a), access to and control over productive capital (assets other than land and pond), the survey captures, fishing/aquaculture related aspects, specifically large fish, small fish, fish producing equipment, fish processing equipment, fish storage equipment, fish transportation equipment and fishing and harvesting equipment. In addition, module 9, we have attitude scale for gender in the context of fisheries and aquaculture; this module asks several fisheries specific questions, such as a man is less of a man if his wife is involved in fish farming; men should be responsible for marketing fish, not women, among others.

This paper implemented the pro-WEFI in order to determine how gender equity in, for instance, access to and control over productive assets (land, pond, fish inputs such as high-quality fish seeds, harvesting equipment, among others) is different and/or similar for women and men in a household that practices aquaculture in northwestern Bangladesh.

Following the background section, the methodology provides detailed description of the pro-WEFI, the domains of empowerment that make up the aggregated index and explanation on the index construction. The findings section indicates the level of empowerment of women in the aquaculture sector relative to men in Bangladesh. Finally, is the conclusion, the results are further discussed and recommendations to advance women in the aquaculture sector are provided.

3. Methodology

The Pro-WEFI instrument was implemented through the IDEA project in Bangladesh. The IDEA project was carried out in Rajshahi and Rangpur divisions (North-western Bangladesh), with the purpose of enhancing the incomes, diets and nutrition of smallholder aquaculture involved families, as well as to increase opportunities for women's empowerment through aquaculture in Bangladesh. Informed consent was sought to the participants of the study before administering the questionnaire. To protect the respondents' anonymity, all study part[illegible]ant names have been replaced with pseudonyms. The authors of this manuscript do not have access to information that could identify individual participants during or after the data collection.

3.1 Data collection

The study was done in two phases. The first phase was a cognitive testing of the questionnaire and the second phase was the actual implementation of the pro-WEFI questionnaire. Below we start by explaining the cognitive testing of the tool and then we follow by explaining the actual implementation of the pro-WEFI tool.

3.1.1. Cognitive testing

The Pro-WEFI was cognitively tested in communities that speak Bengali, in West Bengal, India. The data were collected throughout the month of September 2021. The testing entailed administering survey questions while gathering additional responses on the survey to improve the quality of responses and obtain the intended information. It was conducted by interviewing survey respondents to explore the four stages of the cognitive process in answering the survey questions, which are 1) comprehension, 2) retrieval, 3) judgement, and 4) response. The cognitive testing was conducted with 52 respondents, which comprised 14 men and 38 women from dual-headed households and 4 women from female-headed households. The fieldwork was conducted in three agro-climatic zones in West Bengal, India. The respondents and communities were chosen to be representative of a variety of aquaculture and fisheries

contexts. All of the interviews were conducted in Bengali, and all of the respondents were interviewed alone (i.e., without the presence of other family members or neighbours). The cognitive testing of the pro-WEFI tool only used structured household questionnaire survey, deployed for a man and a woman of the household (husband and wife) who practices aquaculture.

Out of the 12 modules in the Pro-WEFI, presented in this paper, eleven were cognitively tested. The module that was not tested is the time use module, because it was extensively tested and revised as part of WEAI cognitive testing and piloting, including in Bangladesh. Upon completion of the cognitive testing, the tool was revised by removing or modifying questions with high levels of cognitive difficulties. The revised tool was then validated in Bangladesh in 2022. More information about cognitive testing, can be found in McDougall et al., 2022.

3.1.2 pro-WEFI implementation

The study employed a structured Pro-WEFI household survey. In terms of sampling, the study was conducted from July through September of 2022 in the Bogura, Naogaon, and Rangpur divisions of Bangladesh. 217 women and 211 men were interviewed from a total of 217 households in 30 villages engaged in fish production activities. The respondents interviewed as part of the pro-WEFI implementation study included: (*i*) men and women beneficiaries of the IDEA project's nutrition, aquaculture, and gender training; (*ii*) landless women beneficiaries of the IDEA project's aquaculture training; and a (*iii*) control group. **Figure 1** provides an overview of the domains of the pro-WEFI.

[Insert Figure 1]

3.2 Data analysis

The collected datasets were analysed using STATA software version 16. The analysis is based on the domains and indicators of pro-WEFI. Pro-WEFI quantifies women's empowerment status in the aquaculture and fisheries by assessing women's agency using the quantitative survey instrument adapted from the pro-WEAI questionnaire. For the definitions and adequacy cut-offs for every indicator that was applied to calculate the cognitively tested pro-WEFI index (please refer to McDougall et al., 2022). Each indicator is made up of various questions that are given to the interviewed women and men that are included in the study. Finally, a weighted index is calculated using twelve indicators, each indicator received a weight of 1/12, following the pro-WEAI method of calculation.

The composition of pro-WEFI retains important properties from the Pro-WEAI by assessing agency of women and men in the fisheries and/or aquaculture across three key domains: intrinsic agency, which implies power within, instrumental agency, which can also be referred to as power to, and collective agency, which is also recognized as power with (Malapit et al., 2019). Pro-WEFI methodology builds on all 12 indicators from pro-WEAI by adding and updating existing questions with fisheries and aquaculture specific scenarios and options, as partly noted earlier in the background section.

Intrinsic agency consists of four indicators, including self-efficacy, autonomy in income, respect among household members and attitudes about IPV against women. Pro-WEFI includes new scenarios and statements for assessing *self-efficacy* and *attitudes toward IPV against women* compared to pro-WEAI, with subsequent adjustments made to the adequacy cut-offs to

be theoretically consistent. Such changes from pro-WEAI have been made based on the outcomes of the cognitive test conducted prior to the survey.

The instrumental agency in pro-WEFI is composed of six indicators, namely ownership of land, access to and decisions on financial assets, input in productive decisions, work-life balance, control over use of income, visiting important locations. Pro-WEFI adds key decisions pertaining to fishing and post-fishing activities to the list of work activities used in pro-WEAI to assess respondent's *input in productive decisions* and *control over income*. Subsequently, the *ownership of land and assets* indicator have been revised to account for ownership of ponds and access to gleaning area, which are essential resources to households engaged in fisheries and aquaculture. Finally, collective agency is composed of two indicators assessing membership in influential groups and group membership. Pro-WEFI expands on the list of groups questioned on by adding groups that households who participate in fishing and post fishing activities are likely to associate with. Such modifications allow pro-WEFI methodology to track and monitor the various dimensions of empowerment of women participating in the aquaculture and fisheries production and diagnose the impediments to their agency, in an approach to provide better targeted support.

Index construction: responses from the pro-WEFI survey is then used to categorize men and women as adequate or inadequate for all indicators using the corresponding thresholds exhibited in McDougall et al., (2022). Thus, 12 binary variables are deduced, with 0 representing inadequacy in the indicator and 1 indicating adequacy. A weighted average of all indicators, using equal weights of 1/12, is then computed to compute individual empowerment scores for both women and men sampled. Following pro-WEAI, respondents scoring 0.75 or higher are classified as empowered, and conversely, as disempowered if scores below 0.75, i.e., found inadequate in 4 or more out of the 12 pro-WEFI indicators.

Pro-WEFI, following the pro-WEAI methodology, is measured as a weighted mean of two sub-indices. They are the three Domains of Empowerment Index (3DE) and Gender Parity Index (GPI), where the former receives a 90% weight, and the latter is assigned remaining 10%. The *3DE score* is the aggregated individual scores, which measures the respondents' empowerment across three domains. It ranges from 0 to 1, with higher scores representing greater empowerment. GPI is computed using individual scores of the man and woman from the same household to account for the extent of gender equality in the final pro-WEFI index. More information on how the individual indicators are joined together to calculate the 3DE, GPI and composite index can be found in Malapit et al., (2019).

4. Results

4.1 Demographic characteristics

Table 1 exhibits summary statistics of the population sample that participated in the study. The structured survey was administered in 217 households in Bogura, Naogaon, and Rangput districts of Northwestern Bangladesh. All households surveyed were dual adult households, composed of at least one male and one female above eighteen years of age. Nearly two-thirds of the women surveyed had an age range between 26 and 45 years, and on average were younger than the male respondents. Less than 10 percent of both men and women never attended school. For those who completed primary school, 45 percent were women and 36 percent were men. Finally, most men and women in the sample reported being married and identified themselves as Muslims.

[Insert Table 1]

4.2 Aggregate pro-WEFI scores

Table 2 summarizes key results from the aggregate indices. The aggregate pro-WEFI score for women is 0.79, which is a weighted mean of the women's 3DE score of 0.78 and the GPI score of 0.93. The 3DE score implies the achievement of women in the sample across the twelve

indicators, and it integrates both the headcount ratio of women who are disempowered and the intensity of their disempowerment (Malapit et al., 2019).

45 percent of the sampled women and 49 percent of the men attained scores of 0.75 and above, classified as achieving empowerment. The intensity of disempowerment for both women and men are reflected by the mean disempowerment score (1-3DE), which suggests that men and women who had scores below 0.75 failed to achieve adequacy on average in nearly 40 percent of the indicators. 67 percent of women achieved gender parity; the remaining 33 percent of the women had lower empowerment scores on average than the men surveyed from the same households. The average percentage shortfall in empowerment scores for those women who do not have gender parity was 0.22 relative to the men.

[Insert Table 2]

Even though the aggregate pro-WEFI index, 3DE and GPI scores are all valuable ways to aggregate empowerment at the project level for both men and women, making inferences to these high-level indexes together with the sub-indicators, and subcomponents give more well-rounded insights to support women in the fisheries or aquaculture sector, they still do not provide a clear picture of where one needs to focus in advancing gender equality. Thus, the decomposability of the pro-WEFI into the dimensions and indicators, allows the identification of the drivers of change, areas of improvement, and the gendered difference in the adequacy achievements across different types of agency and indicators.

4.3 Uncensored and censored headcount ratios

Table 3 shows results of the uncensored and censored headcount ratios of inadequacy for the 12 pro-WEFI indicators by gender. Uncensored headcount ratios reflect the percent of respondents found inadequate in a given indicator, irrespective of their overall empowerment status. Whereas the censored headcount reflects the percent of interviewee who are classified as disempowered, and also are inadequate in a given indicator, as defined by Alkire and Foster (2011). Since nearly half of both women and men in the sample are disempowered, the figures in censored and uncensored headcounts reflect differences.

[Insert Table 3]

Most women in the sample reported inadequacy in their ability to visit important locations (79 percent). Similarly, most women were inadequate in having access to or making decisions on financial services (56 percent). However, women reported very high adequacies in having input in decision making. This was not the case for the landless women, however, who reported being the primary decision maker in their household, as they were the only earning member in the family. In terms of ownership of other assets and land, the findings indicated very high adequacies. Similarly, high adequacy scores were reported in achieving work balance.

Intriguingly, a higher percentage of men compared to the women are found inadequate across most indicators under intrinsic and collective agency, namely, respect among household members, autonomy in income, attitudes towards IPV against women, group membership and membership in influential groups. The existence of groups, especially influential ones were very limited and most of the groups that do exist are micro-credit groups where women are members. However, men in the sample fared better on average in achieving adequacy in the instrumental agency indicators with the exception of work balance.

It is important to compare the censored and uncensored headcount ratios to understand the inadequacy of indicators in relation to the status of empowerment. For women, the censored and uncensored ratios are very similar for attitudes about IPV against women, input in productive decisions, ownership of land and assets, and work balance, which suggest that most women who are inadequate in these three indicators are disempowered.

4.4 Distribution of inadequacies by sex

Figure 2 compares the distribution of the number of inadequate indicators across genders. Respondents with 4 or more inadequate indicators out of the 12 (i.e. with an individual score below 0.75 out of 1) are classified as disempowered, represented by the shaded area in Fig 2. For [illegible]he disempowered individuals, number of average inadequacies of the men and women in the sample are very similar as we find both distributions to be mostly overlapping in the shaded area. Fig 2, shows that the intensity of disempowerment experienced by men and women in the sample are very comparable, as both distributions in the figure follow a very similar pattern for 4 or more inadequacies. This observation is line with the results exhibited in the high-level indices summary in table 3, where both headcount of respondents not achieving empowerment and the average disempowerment score for women are nearly the same as that of men, suggesting similar intensity of disempowerment.

4.5 Empowerment and agency of women

One of the key objectives of developing pro-WEFI is to identify the key obstacles facing the empowerment and agency of women who engage in fisheries or aquaculture. The diagnostics presented in **Figure 3** and table 3 provide key insights in highlighting the highest contributors to the disempowerment of the women in the sample. Figure 3 displays the absolute contribution of each of the 12 pro-WEFI indicators, represented by the different coloured bars, to the

disempowerment score (1-3DE) of the sampled women and men. The total disempowerment scores, 0.22 for women and 0.2 for men, are reflected in the depth of each bar in the figure.

[Insert Figure 3]

Table 4 exhibits the proportional contribution of each indicator to the disempowerment of men and women respondents who have not yet achieved empowerment. For ways of computing see (Malapit et al., 2019). Such a diagnostic helps identify the key challenges facing the disempowered in the sample. For women, the biggest impediments to their achievement of empowerment comes from the lack of access to and decision making on financial services (15 percent), inability to visit important locations (19 percent) and lack of membership in influential groups (15 percent) and any groups in general (14 percent). We also find group membership and membership in influential groups making significant contributions to the disempowerment of men in the sample. Additionally, lacking autonomy in the use of income and respect among household members pose threat to men's achievement of empowerment.

[Insert Table 4]

4.6 Intrahousehold patterns of empowerment

The sample from the pilot study of pro-WEFI comes from all dual adult households, where both women and men form the same household self-report their adequacies or inadequacies across several indicators. This allows us to examine the intrahousehold patterns of empowerment by comparing their individual weighted average scores, using the pro-WEFI quantitative methodology discussed earlier. Among the 208 households, that had responses from both women and men, we find that 43.5 percent had men being adequate in more indicators than women (**Table 5**). One-fifth of the surveyed households had the woman and

the man adequate in equal number of indicators, and 36 percent had women adequate in more indicators than men. We also find that only in 21 percent of the overall sample, both men and women are empowered, and in 27 percent of the household neither the woman nor the man achieved empowerment. Intriguingly, the data shows that only the woman of the household is empowered in about a quarter of the households that were surveyed.

[Insert Table 5]

4.7 Robustness

4.7.1 Association between pro-WEFI indicators

As discussed earlier, each indicator in the pro-WEAI, and therefore pro-WEFI, methodology is assigned an equal weight of 1/12. It is therefore a useful exercise to assess the pairwise associations between each indicator. A high association between two indicators can imply more weights assigned to an indicator pair (Malapit et al., 2019). We use Cramer's V to identify the strength of the correlation, where two variables are weakly correlated if Cramer's V is less than 0.3. Most pro-WEFI indicators are weekly correlated it with each other, with a few exceptions (**Table 6**). A slight correlation is observed between autonomy and control over income (Cramer's V=0.56), which may suggest that although the indicators of autonomy and control over income are distinct, they are likely to be related. We also find strong correlation between group membership and membership in influential groups (Cramer's V=0.82) because it is a necessary condition to be adequate in the former to be adequate in the latter. Malapit et al. (2019) makes similar observations studying the associations between pro-WEAI indicators and explains that the choice to define the said indicators was deliberate to assign more weights to the collective agency which has very fewer indicators compared to the other two agencies.

[Insert Table 6]

4.7.2 Redundancy between Pro-WEFI indicators

We further analyse the redundancy between pro-WEFI indicators to examine how inadequacies are clustered. **Table 7** shows the redundancy between the 12 indicators, calculated using Alkire et al., (2015) definition of redundancy. We find high redundancy among all pro-WEFI indicators, as also observed by Malapit et al., (2019) with pro-WEAI indicators. Since we mostly observe weak associations among indicators (Table 7), high redundancy does not pose major risk to the robustness of the weighted pro-WEFI index. However, high values of redundancy among indicators are suggestive that inadequacies are grouped.

[Insert Table 7]

4.8 Sensitivity analysis

We observed how the aggregate 3DE scores and the percent of respondents achieving empowerment for men and women vary by considering (1) different empowerment cut-offs and (2) an alternative weighing scheme, following Alkire et al., (2015).

Table 8 exhibits the percent of men and women achieving empowerment across a range of empowerment cut-offs. Each column represents a different cut-off, i.e. adequacy in number of indicators required, to be classified as achieving empowerment. 0.75 cut-off used for pro-WEFI methodology, following pro-WEAI, represents achieving adequacy in 9 out of 12 indicators. The results suggest that across all cut-offs considered, higher percent of men achieve empowerment compared to women, i.e., men rank higher than women. Thus, the rank robustness results in table 9 provide no evidence for altering the empowerment cut-off for pro-WEFI from 0.75.

[Insert Table 8]

Next, we compare 3DE scores and headcount ratio of empowerment for men and women considering two different weighing schemes: (1) 3DE score computed using equal weights for all indicators, followed by pro-WEFI (2) 3DE score computed using equal weights for three domains. Th second scheme assigns a weight of 1/3 for each of the three domains used in pro-WEFI (Table 1), namely intrinsic, instrumental and collective agency, and the indicators within each domain receive equal weights. Thus, the indicators within (1) intrinsic agency receive a weight of 1/12 each, (2) instrumental agency receive a weight of 1/18 and (3) collective agency receive a weight of 1/6. **Table 9** compares 3DE scores and headcount ratio for men and women, and assigns ranks accordingly. Under the existing pro-WEFI scheme, i.e., scheme 1, men rank higher than women with a higher 3DE score (0.8) and a higher percent achieving empowerment (80%) compared to women, and thereby secure rank 1. However, the ranking changes under the alternative scheme, where more women achieve empowerment (52%) with a higher aggregate 3DE score of 0.76. The results change noticeable for two main reasons. First, the biggest contributors to women's disempowerment, i.e., access to and decisions on credit and ability to visit important locations, receive much lower weights (1/18) under scheme 2, compared to scheme 1 (1/12). This increases the aggregate empowerment score of women, together with their headcount ratio for those achieving empowerment. Conversely, biggest contributors to men's empowerment, namely group membership and membership in influential group, receive double the weights under scheme 2 (1/6) compared to scheme 1 (1/12), lowering the aggregate empowerment score and the headcount ratio for men. Therefore, under scheme 2 the difference between result for men and women grow higher, in favour of the latter. We retain scheme 1 as the chosen weighting scheme for pro-WEFI for two key reasons: 1) The

ranking of men and women's scores and headcount ratio observed under scheme1 aligns with findings from other similar research which show that men have higher empowerment scores than women in Bangladesh (Alkire et al., 2013) and (Ahmed et al., 2018); 2) The number of indicators is not balanced across the three domains, which would assign a heavier weight to the collective agency indicators compared to the instrumental and intrinsic agency indicators (Malapit et al., 2019). Because we have no theoretical basis for weighting some indicators higher than others, we have decided to give all indicators the same weight, which will further allow us comparability across various projects targeted at respondents engaged in fisheries and/or aquaculture.

[Insert Table 9]

5.Conclusion

There is ample evidence endorsing the need to engage and empower women in fisheries and aquaculture, whose role in this sector contributes significantly to household's income and food security (UN Women, 2020). This is so, because women's contribution to aquaculture is under-recognized and largely seen as an extension of their household chores (Githukia et al., 2020), it results to their exclusion from decision making in the process (Choudhury et al., 2017; Torre et al., 2019; UN Women, 2020). In this study we present pro-WEFI methodology and results from a pilot study conducted to quantify the empowerment and agency of women engaged in aquaculture. We used a survey-based data from 217 households engaged in fish production activities from several districts in Bangladesh, to devise the composite index, pro-WEFI. The methodology and results presented in this paper is based on the cognitively tested survey

instrument, where men and women were asked to self-report the extent of their decision making, agency and access to various resources, across three key domains. We find nearly one-third of the sampled households not achieving gender parity. Even though we find the mean empowerment score of men to be higher than that of women, the mean 3DE score of women we have found is higher than other studies measuring empowerment of women in Bangladesh. One key reason for such observation is because of the selection bias in this study, where we surveyed women already engaged in fish production activities, who inherently are more expected to be empowered than average women.

The decomposability of pro-WEFI into individual indicators further allows us to identify where women face more challenges and areas of improvement to augment women's empowerment, specifically in the aquaculture sector. Our results show that, for the sampled women, inability to visit important locations, lack of ownership of land and other assets, lack of access to credit, not being a member to influential groups are most disempowering aspects for women in the household that practices aquaculture. These findings show that the in-depth assessments of the pro-WEFI and its domains and indicators can help projects to: a) better design interventions to create targeted impact on critical domains, and b) see how adequacy across different indicators change after an intervention. The findings show that there is a huge need to address the social and gender norms that hinder women from participating in the markets (mobility constraints), owning assets, among other gender norms. Whether women will be permitted by their spouse to visit was based on their ability to fulfil household duties and maintaining *purdah* (covering veil) (Froukje et al., 2022). Addressing these deeply embedded power imbalances and discriminatory norms requires a gender transformative approach (GTA) with an intersectional lens—one that goes beyond the male-female binary to tackle the multiple layers of social inequalities that exclude some women and men more than others. Such an approach also engages the historically more powerful groups i.e. men and boys, and formal public and private

actors in re-negotiating for more equal access and control of resources, decision-making power and benefits from development interventions.

Declaration of Competing Interest

The authors declare that they have no competing financial interests or personal relationships that could have appeared to influence the research work reported in this manuscript.

Ethics statements

This study was performed in line with the principles of the Declaration of Helsinki. Approval was granted by the Ethics Committee of the University of Dhaka, Institute of Health Economics.

Data availability statement

Data is available upon request.

Acknowledgements

This research has been carried out as part of the Aquaculture: Increasing Income, Diversifying Diets, and Empowering Women in Bangladesh and Nigeria (IDEA) project number INV009865. The financial support for this study was provided by the Bill & Melinda Gates Foundation. In addition, this work was also carried out under the CGIAR GENDER Impact Platform, which is grateful for the support of CGIAR Trust Fund contributors: www.cgiar.org/funders. We also would like to thank the Resilient Aquatic Food Systems for Healthy People and Planet (RAqFS) One CGIAR research and development initiative for providing the platform to carry out this study. In addition, we thank the Ecociate Consultants Pvt. Ltd for collecting data. In addition, we thank Dr. Colin Shelley and Alvaro Paz Mendez for providing the leadership in the IDEA project.

References

Ahmed, Tanima, and Binayak Sen. 2018. "Conservative Outlook, Gender Norms and Female Wellbeing: Evidence from Rural Bangladesh." *World Development* 111: 41–58. https://doi.org/10.1016/j.worlddev.2018.06.017.

Alkire, Sabina et al. 2013a. The Women's Empowerment in Agriculture Index *The Women's Empowerment in Agriculture Index*. http://ophi.qeh.ox.ac.uk/.

———. 2013b. "The Women's Empowerment in Agriculture Index." *World Development* 52: 71–91.

———. 2015. Reflexive Research and the (Re)Turn to the Baroque *Data and Analysis*.

Alkire, Sabina, and James Foster. 2011. "Counting and Multidimensional Poverty Measurement." *Journal of Public Economics* 95(7–8): 476–87. http://dx.doi.org/10.1016/j.jpubeco.2010.11.006.

Anik, Asif Reza, and Sanzidur Rahman. 2021. "Women's Empowerment in Agriculture: Level, Inequality, Progress, and Impact on Productivity and Efficiency." *Journal of Development Studies* 57(6): 930–48.

Bayeh, Endalcachew. 2016. "The Role of Empowering Women and Achieving Gender Equality to the Sustainable Development of Ethiopia." *Pacific Science Review B: Humanities and Social Sciences* 2(1): 37–42. http://dx.doi.org/10.1016/j.psrb.2016.09.013.

Bayissa, Fitsum, Jeroen Smits, and Ruerd Ruben. 2018. "The Multidimensional Nature of Women's Empowerment: Beyond the Economic Approach." *Journal of International Development* 30(4): 661–90.

Choudhury, Afrina, Cynthia McDougall, and Surendran Rajaratnam. 2017. *Women's Empowerment in Aquaculture Two Case Studies from Bangladesh*. https://www.google.com/url?sa=t&rct=j&q=&esrc=s&source=web&cd=&cad=rja&uact=8&ved=2ahUKEwiL0MO5tOL8AhW2TKQEHVB_DeEQFnoECAgQAQ&url=http%3A%2F%2Fpubs.iclarm.net%2Fresource_centre%2F4160.pdf&usg=AOvVaw3LVBjH1Hdyiis29tBEw_5z.

Colverson, Kathleen Earl et al. 2020. "Evolution of a Gender Tool: WEAI, WELI and Livestock Research." *Global Food Security* 26(April): 100375. https://doi.org/10.1016/j.gfs.2020.100375.

Diiro, Gracious M. et al. 2018. "Women's Empowerment in Agriculture and Agricultural Productivity: Evidence from Rural Maize Farmer Households in Western Kenya." *PLoS ONE* 13(5): 1–27.

Githukia, Cecilia Muthoni et al. 2020. "African Journal of Agricultural Research Gender Roles and Constraints in the Aquaculture Value Chain in Western Kenya." 16(5): 732–45. http://www.academicjournals.org/AJAR.

Heckert, Jessica, Deanna K. Olney, and Marie T. Ruel. 2019. "Is Women's Empowerment a Pathway to Improving Child Nutrition Outcomes in a Nutrition-Sensitive Agriculture Program?: Evidence from a Randomized Controlled Trial in Burkina Faso." *Social*

Science and Medicine 233(April): 93–102. https://doi.org/10.1016/j.socscimed.2019.05.016.

Klasen, Stephan. 2006. "UNDP's Gender- related Measures: Some Conceptual Problems and Possible Solutions." *Journal of Human Development* 7(2): 243–74.

Malapit, H. et al. 2019. "Development of the Project-Level Women's Empowerment in Agriculture Index (pro-WEAI)." *World Development* 122: 675–92. https://doi.org/10.1016/j.worlddev.2019.06.018.

Malapit, Hazel Jean L et al. 2015. "Women ' s Empowerment Mitigates the Negative Effects of Low Production Diversity on Maternal and Child Nutrition in Nepal." *The Journal of Development Studies* 51(8): 1097–1123. http://dx.doi.org/10.1080/00220388.2015.1018904.

Mganga, Andrew Evarist et al. 2021. "Development of a Women's Empowerment Index for Tanzania from the Demographic and Health Surveys of 2004–05, 2010, and 2015–16." *Emerging Themes in Epidemiology* 18(1): 1–11. https://doi.org/10.1186/s12982-021-00103-6.

Miedema, Stephanie Spaid, Regine Haardörfer, Amy Webb Girard, and Kathryn M. Yount. 2018. "Women's Empowerment in East Africa: Development of a Cross-Country Comparable Measure." *World Development* 110: 453–64. https://doi.org/10.1016/j.worlddev.2018.05.031.

Mobarok, Mohammad Hasan, Theodoros Skevas, and Wyatt Thompson. 2021. "Women's Empowerment in Agriculture and Productivity Change: The Case of Bangladesh Rice Farms." *PLoS ONE* 16(8 August): 1–21. http://dx.doi.org/10.1371/journal.pone.0255589.

Narayanan, Sudha et al. 2019. "Developing the Women ' s Empowerment in Nutrition Index in Two States Of." *Food Policy* (June): 101780. https://doi.org/10.1016/j.foodpol.2019.101780.

Parrao, Constanza Gonzalez et al. 2021. "Aquaculture for Improving Productivity, Income, Nutrition and Women's Empowerment in Low- and Middle- income Countries: A Systematic Review and Meta- analysis." *Campbell Systematic Reviews* 17(4).

Quisumbing, Agnes, Ruth Meinzen-Dick, and Hazel Malapit. 2021. "Women's Empowerment and Gender Equality in South Asian Agriculture: Measuring Progress Using the Project-Level Women's Empowerment in Agriculture Index (pro-WEAI) in Bangladesh and India." *World Development* (xxxx): 105396. https://doi.org/10.1016/j.worlddev.2021.105396.

Rahman, M. Hammadur, and Kazi Naoroze. 2007. "Women Empowerment through Participation in Aquaculture: Experience of a Large-Scale Technology Demonstration Project in Bangladesh." *Journal of Social Sciences* 3(4): 164–71.

Rettig, Erica M., Stephen E. Fick, and Robert J. Hijmans. 2020. "The Female Empowerment Index (FEMI): Spatial and Temporal Variation in Women's Empowerment in Nigeria." *Heliyon* 6(5): e03829. https://doi.org/10.1016/j.heliyon.2020.e03829.

Saha, Shree, and Sudha Narayanan. 2022. "A Simplified Measure of Nutritional Empowerment : Using Machine Learning to Abbreviate the Women ' s Empowerment in Nutrition Index." *World Development* 154: 105860.

https://doi.org/10.1016/j.worlddev.2022.105860.

Teufel, A Galiè N, L Korir I Baltenweck, and A Webb Girard. 2019. "The Women ' s Empowerment in Livestock Index." *Social Indicators Research* 142(2): 799–825. https://doi.org/10.1007/s11205-018-1934-z.

Torre, Jorge et al. 2019. "Women's Empowerment, Collective Actions, and Sustainable Fisheries: Lessons from Mexico." *Maritime Studies* 18(3): 373–84.

UN Women. 2020. *Women's Economic Empowerment in Fisheries in the Blue Economy of the Indian Ocean Rim - A Baseline Report*. NewYork. https://www.unwomen.org/en/digital-library/publications/2020/10/womens-economic-empowerment-in-the-indian-ocean-rim.

Worsdale, Rosie, and Jack Wright. 2021. "My Objectivity Is Better than Yours: Contextualising Debates about Gender Inequality." *Synthese* 199(1–2): 1659–83. https://doi.org/10.1007/s11229-020-02835-5.

Yount, Kathryn M. et al. 2019. "Measurement Properties of the Project-Level Women's Empowerment in Agriculture Index." *World Development* 124(January).

Tables

Table 1. Sample characteristics of respondents by sex

Variable	Percent of respondents	
	Women	Men
Number of observations	217	208
Age group		
16-25	9.2	4.3
26-45	63.1	47.1
46-65	27.2	45.7
>65	0.5	2.9
Education		
Never attended school	6	7.7
Below Primary	17.1	11.1
Primary	45.2	35.6
Secondary	16.6	22.1
Higher secondary or above	15.2	23.6
Marital Status		
Never married	0.5	3.8
Married	98.6	96.2
Divorced/Separated/Widowed	0.9	0
Religion		
Islam	88.5	88
Hinduism	11.5	12
Dual Adult Household	100	100

Note: Weighted by inverse project sample size.

Table 2. Pro-WEFI scores, by sex of respondent

Indicator	Women	Men
Number of observations	217	208
**3DE score**	**0.78**	**0.80**
Disempowerment score (1 – 3DE)	0.22	0.20
% Achieving empowerment	45%	49%
% Not achieving empowerment	55%	51%
Mean 3DE score for not yet empowered	0.60	0.61
Mean disempowerment score (1 – 3DE)	0.40	0.39
**Gender Parity Index (GPI)**	**0.93**	
Number of dual-adult households	208	
% Achieving gender parity	67%	
% Not achieving gender parity	33%	
Average empowerment gap	0.22	
**Pro-WEFI score**	**0.79**	

Table 3. Headcount ratios of inadequacy in Pro-WEFI indicators

	Uncensored headcount (ratio%)		Censored headcount (ratio%)	
	Women	Men	Women	Men
Intrinsic agency				
Autonomy in income	36.4	42.3	23.5	31.7
Self-efficacy	38.2	11.5	25.8	7.7
Attitudes about intimate partner violence against women	2.3	2.9	1.4	2.4
Respect among household members	16.6	36.5	12.0	27.4
Instrumental agency				
Input in productive decisions	5.5	2.9	5.1	2.4
Ownership of land and other assets	1.4	0.5	1.4	0.5
Access to and decisions on financial services	56.2	34.1	41.0	25.0
Control over use of income	38.7	31.3	24.4	26.0
Work balance	7.8	25.0	6.0	15.4
Ability to visit important locations	78.8	8.2	49.8	3.8
Collective agency				
Group membership	42.9	58.2	36.9	49.5
Membership in influential groups	48.4	72.6	39.6	50.0

Table 4. Relative contributions of each indicator to disempowerment

	Proportional contribution to disempowerment (%)	
	Women	Men
Intrinsic agency		
Autonomy in income	8.8	13.1
Self-efficacy	9.7	3.2
Attitudes about intimate partner violence against women	0.5	1.0
Respect among household members	4.5	11.3
Instrumental agency		
Input in productive decisions	1.9	1.0
Ownership of land and other assets	0.5	0.2
Access to and decisions on financial services	15.4	10.3
Control over use of income	9.2	10.7
Work balance	2.2	6.4
Ability to visit important locations	18.7	1.6
Collective agency		
Group membership	13.8	20.5
Membership in influential groups	14.9	20.7

Table 5. Intrahousehold patterns of empowerment

	% of dual-adult households
Male adequacy score > female adequacy score	43.53
Female adequacy score > male adequacy score	35.76
Female adequacy score = male adequacy score	20.71
Total	100.00
Only male is empowered	27.40
Only female is empowered	24.04
Both male and female are empowered	21.15
Neither male nor female are empowered	27.40
Total	100.00

Table 6. Association (Cramer's V) between Pro-WEFI indicators.

	Autonomy in income	Self-efficacy	Attitudes about domestic violence	Respect among household members	Input in productive decisions	Ownership of land and other assets
Intrinsic agency						
Autonomy in income	1.000					
Self-efficacy	-0.256	1.000				
Attitudes about intimate partner violence against women	-0.040	0.042	1.000			
Respect among household members	-0.197	0.108	0.104	1.000		
Instrumental agency						
Input in productive decisions	-0.002	0.040	-0.034	-0.020	1.000	
Ownership of land and other assets	-0.078	0.000	-0.016	-0.003	-0.020	1.000
Access to and decisions on financial services	0.098	0.081	0.030	-0.052	0.019	0.009
Control over use of income	0.560	-0.165	0.036	-0.115	0.262	-0.021
Work balance	0.051	-0.050	-0.032	-0.003	-0.029	-0.043
Ability to visit important locations	-0.154	0.302	0.004	-0.103	0.071	0.060
Collective agency						
Group membership	0.047	0.088	0.014	0.348	0.162	0.048
Membership in influential groups	-0.104	0.061	-0.019	0.268	0.123	0.079
	Access to and decisions on financial services	Control over use of income	Work balance	Visiting important locations	Group membership	Membership in influential groups

Instrumental agency						
Access to and decisions on financial services	1.000					
Control over use of income	0.172	1.000				
Work balance	-0.132	0.011	1.000			
Ability to visit important locations	0.130	-0.108	-0.264	1.000		
Collective agency						
Group membership	0.036	-0.050	0.054	-0.054	1.000	
Membership in influential groups	0.036	-0.149	0.084	-0.119	0.818	1.000

	Autonomy in income	Self efficacy	Attitudes about domestic violence	Respect among HH members	Input in productive decisions	Ownership of land and other assets
Intrinsic agency						
Autonomy in income	1.000					
Self-efficacy	0.659	1.000				
Attitudes about intimate partner violence against women	0.969	0.978	1.000			
Respect among household members	0.667	0.776	0.984	1.000		
Instrumental agency						
Input in productive decisions	0.957	0.962	0.973	0.955	1.000	
Ownership of land and other assets	0.984	0.991	0.990	0.990	0.990	1.000
Access to and decisions on financial services	0.651	0.780	0.978	0.716	0.961	0.991
Control over use of income	0.864	0.696	0.978	0.699	0.996	0.989
Work balance	0.853	0.827	0.972	0.837	0.955	0.989
Ability to visit important locations	0.540	0.865	0.975	0.696	0.970	0.996
Collective agency						
Group membership	0.630	0.787	0.976	0.891	0.991	0.995

Table 7. Redundancy between Pro-WEFI indicators

Membership in influential groups	0.544	0.781	0.970	0.882	0.988	1.000
	Access to and decisions on financial services	Control over use of income	Work balance	Visiting important locations	Group membershi p	Membership in influential groups
Instrumental agency						
Access to and decisions on financial services	1.000					
Control over use of income	0.724	1.000				
Work balance	0.793	0.841	1.000			
Ability to visit important locations	0.616	0.603	0.751	1.000		
Collective agency						
Group membership	0.564	0.626	0.858	0.531	1.000	
Membership in influential groups	0.568	0.562	0.876	0.485	1.000	1.000

Table 8. Rank comparison of headcount ratios by gender for different empowerment cut-offs

		Number of indicators required to be empowered											
		12	*11*	*10*	*9*	*8*	*7*	*6*	*5*	*4*	*3*	*2*	*1*
Men	Empowerment Headcount ratio	6%	6 %	21 %	49 %	49 %	75 %	98 %	98 %	99 %	100 %	100 %	100 %
	Rank	1	1	1	1	1	1	1	1	1	1	1	1
Women	Empowerment Headcount ratio	0%	0 %	5%	45 %	45 %	73 %	95 %	95 %	98 %	100 %	100 %	100 %
	Rank	2	2	2	2	2	2	2	2	2	2	2	2

Table 9. Rank of 3DE score by gender for different weighing schemes

	Equally weighted by indicator			Equally weighted by domain		
	3DE score	% Achieving empowerment	Rank	3DE score	% Achieving empowerment	Rank
Men	0.80	49	1	0.69	37	2
Women	0.78	45	2	0.76	52	1

Women’s empowerment in fisheries and aquaculture index

Click here to access/download;Figure;Figures.docx

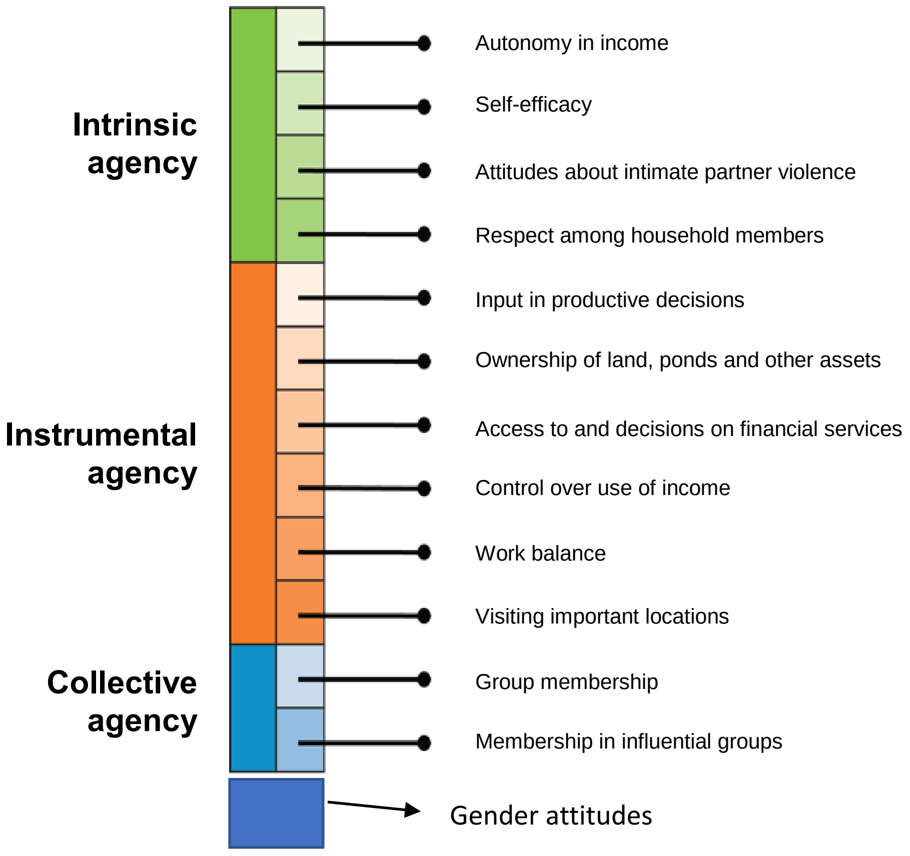

Figure 1. The domains and indicators of Pro-WEFI (Source: McDougall, et al. 2022)

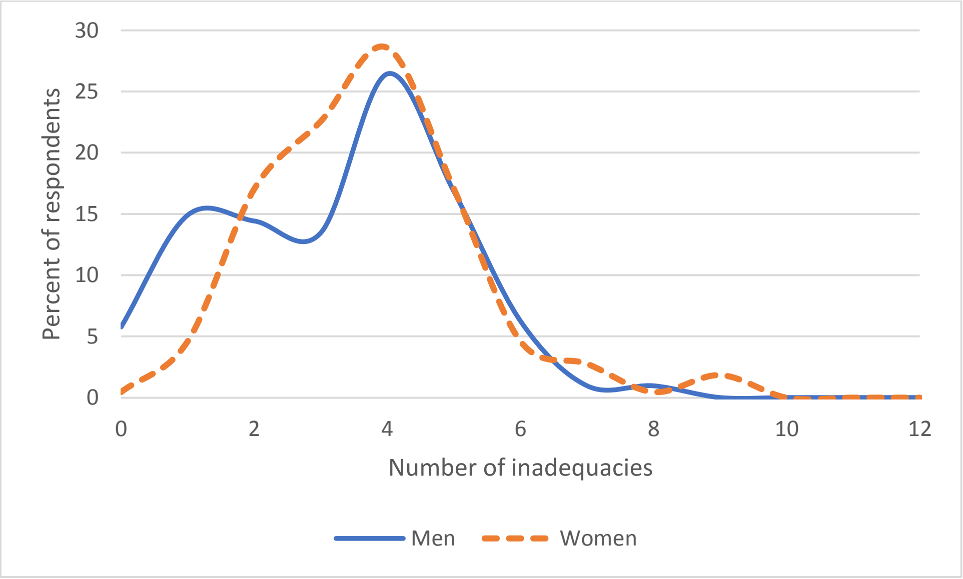

Figure 2. Distribution of inadequacies by sex

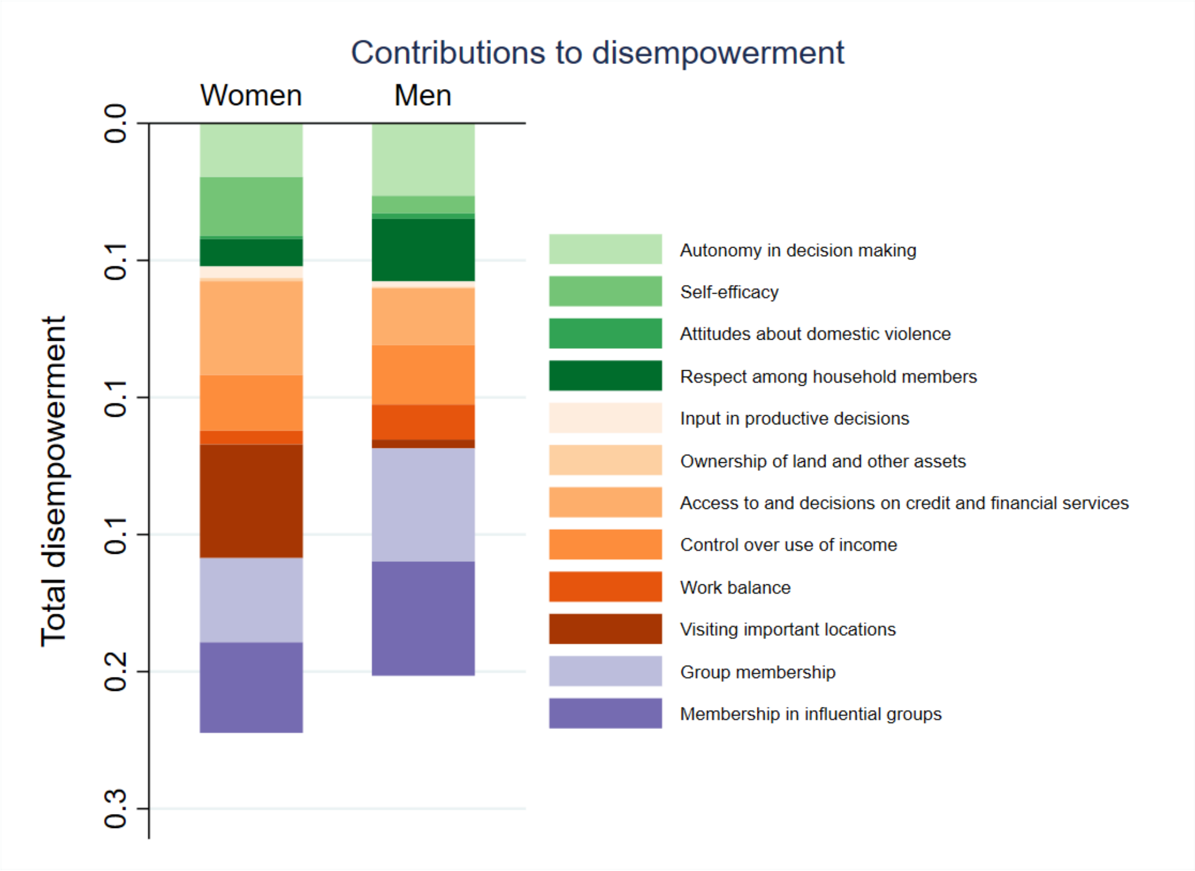

Figure 3. Contributions of each pro-WEFI indicator to disempowerment by sex of the respondents
