# Supplementary material for: Measuring women’s empowerment in aquaculture in northwestern Bangladesh using a project level women’s empowerment in fisheries index (pro-WEFI)
Source: PLoS One. 2025 Jan 10;20(1):e0314284. doi: 10.1371/journal.pone.0314284 (PMC11723601; doi:10.1371/journal.pone.0314284)
Supplement: Attachment — Submitted filename: Pro-WEFI bangladesh response to reviewers 02.10.23.docx [file pone.0314284.s004.docx]

**Response to reviewers’ comments**

| **Reviewer 1 comments** | **Response** |
| --- | --- |
| I recommend that the abstract is revised. | The abstract has been revised |
| This paper does not measure impact. I suspect that this paper presents the results of a baseline that was conducted as part of the IDEA project. I assume that this is why you collected data from three different groups, even though the paper does not present the data per group. I assume that you will make the comparisons when you have the mid or endline data. | The study assessed the impact of the IDEA project on women’s empowerment.  The respondents interviewed as part of the pro-WEFI implementation study included men and women beneficiaries of the IDEA project’s nutrition, aquaculture, and gender training. |
| I suggest that you rewrite the abstract to better reflect what this paper is all about. | The abstract has been re-written |
| In general, the introduction is not well written and should be significantly improved. It is markedly worse than the rest of the paper and seems to have been written by a different person | The introduction has been revised |
| Word choice, this is the second time pivotal is used in this sentence. | Not in the revised introduction |
| Why do you say "on the other hand" here? It does not make sense. | Not in the revised introduction |
| What decision making are they excluded from? Aquaculture management? Value chain related decisions (what species to grow, when to harvest, when to sell)? Etc. | Not in the revised introduction |
| Is it correct to say that the paper implemented the survey? I'd think the implementer was the IDEA project. | Not in the revised introduction |
| Why would you collect names in the first place and why replace it with a pseudonym rather than a number? | This has been corrected and the statement removed from the article |
| It is interesting that you collected data from the different groups of people. How many persons did you interview in each grouping?  Also, you explain here that you interviewed the three groups of people, but you don't present the data by the three groups. All the data is presented as one uniform group and you are not analyzing if there are differences between the three groups. What is the rationale for interviewing three different groups if you do not follow up by analyzing the data to compare the groups? | The respondents interviewed as part of the pro-WEFI implementation study included men and women beneficiaries of the IDEA project’s nutrition, aquaculture, and gender training. |
| I feel that the authors miss an important point here. I assume that they did not expect to find such similar scores between men and women. I believe there are a couple of reasons for this finding  1. Both men and women living in rural Bangladesh societal, governance, and economic hardships. Struggling is not unique to women only. 2. The questions are identifying to what extent men and women feel empowered within the three domains, but it does not ask people to compare the status of women to that of men. It is only asking people to give a perception of their own situation. 3. The questions are getting at to what extent people feel empowered. However, they do not get at power imbalances between men and women, social injustices, and cultural differences between for example how girls and boys are raised. The fact that women are not allowed to go to the market without permission from their husbands is an indication of large power imbalances and social injustice between women and men. | The description of the results has been revised and now includes “Overall, men have fewer inadequacies than women, whose distribution lie slightly to the right of men’s distribution of inadequacies in the shaded area. However, the intensity of disempowerment experienced by men and women in the sample are very comparable, as both distributions in the figure follow a very similar pattern for 4 or more inadequacies. This observation is line with the results exhibited in the high-level indices summary in Table 2, where both headcount of respondents not achieving empowerment and the average disempowerment score for women are nearly the same as that of men, suggesting similar intensity of disempowerment.” |
| I find this impediment problematic because you don't explain the root causes to this until the conclusions. There is so much to unpack in the inability to visit important locations. The fact that women are not empowered to make their own decisions about when and where to go, indicates a deeply embedded lack of empowerment. However, in the paper, it is treated as a shallow factor, almost like a physical inability. | The root causes have been added in the discussion section. |
| You could do a lot more with the conclusions. While you talk about the areas where women are disempowered, what are the strengths in women's empowerment that future aquaculture projects. Also, what are the weaknesses of the tool and what would you do differently - or what follow on studies would you propose to get deeper into this topic? | The weaknesses of the tool have been discussed under the limitations of the study. |
| I don't think this paper leads us to this conclusion. The survey did not give us any information about women's contribution to the aquaculture sector. Furthermore, women in general seemed to score higher than men in "input in productive decisions" financial services decisions" and "control over use of income", while they scored poorly on women's ability to visit important locations. I think this is a case where your data does not line up well with what you thought you would find. | The findings changed after the data analysis was redone. Lack of autonomy in the use of income, was the most disempowering aspect for women.  The conclusions have been revised. |

| **Reviewer 2 comments** | **Response** |
| --- | --- |
| Authors should revise the whole abstract section by having the following unstructured sections: purpose, methodology, results, and conclusion.  "and" and "agency" should be removed from keywords. | The abstract has been revised |
| Introduction needs additional work and should have the following things: 1) Research gap 2) Study objective 3) Research questions or hypotheses 4) Study contribution | The introduction has been revised |
| References throughout the manuscript should be updated and use recent studies from 2018–2023, especially in the introduction section. The following studies might help in this regard: <https://doi.org/10.3390/agriculture12081161> <https://doi.org/10.1016/j.worlddev.2020.105292> | Some of the older references e.g. Rowlands, 1995 give important backgrounds on the topic. |
| "Background and motivation for creating and implementing pro-WEFI" should be changed to review of literature and should include findings of the previous studies | The sub-section "Background and motivation for creating and implementing pro-WEFI" has been removed |
| Hypotheses should also be constructed on the basis of this literature review. | The introduction has been revised to include the hypotheses |
| Also, provide a graphical presentation of the conceptual framework of the study. | Figure 1 comprehensively shows the domains and indicators of Pro-WEFI (Source: McDougall, et al. 2022) |
| Line 161: Why was the survey questionnaire tested in India and not in Bangladesh? Surprising | Since the study was undertaken during covid-19 period with movement restriction , the survey was tested in India as the communities share the same characteristics and agro ecological zones |
| Please add a section called "discussion" to discuss your results thoroughly. I am wondering if a paper can be written without discussing the results in light of previous literature | The discussion section has been added |
| The background statistics presented in Table 1 are not enough to show the background of the respondents. | The table has been revised after data reanalysis |
| The authors should come up with some solid results. Currently, the data has been presented in a vague manner. The authors should draw some conclusions based on their results. | The results, discussion and conclusions sections have been revised |
| Where are the limitations of the study? | The limitations of the study has been included |
| Please also use some statistical models to improve the credibility of your results. | We use Cramer’s V to identify the strength of the correlation between pro-WEFI indicators. |
